# Supplementary material for: The Arabidopsis AtSWEET13 transporter discriminates sugars by selective facial and positional substrate recognition
Source: Commun Biol. 2024 Jun 24;7:764. doi: 10.1038/s42003-024-06291-6 (PMC11196581; doi:10.1038/s42003-024-06291-6)
Supplement: Supplementary file 2 — Supplementary Information [file 42003_2024_6291_MOESM2_ESM.pdf]

Supplemental Information

The Arabidopsis AtSWEET13 transporter discriminates sugars by selective facial and positional substrate recognition

Austin T. Weigle[1] *& Diwakar Shukla*[*2,3,4,5]

[1]Department of Chemistry, [2]Department of Chemical & Biomolecular Engineering, [3]Department of Plant Biology, [4]Department of Bioengineering, [5]Center for Biophysics and Computational Biology

University of Illinois at Urbana-Champaign, Urbana, IL 61801, United States

* E-mail: diwakar@illinois.edu

Table of Contents

Figures and Tables

S. Figure 1 tICA decomposition given simplified MSM features for AtSWEET13 transport processes S-6

S. Figure 2 Scatterplot representation of transport versus the difference in gating highlights that substrate transport proceeds through an hour-glass state S-7

S. Figure 3 AtSWEET13 transport cycles for GLC and SUC translocation implicate minimal conformational change outside of commitment to alternate access S-8

S. Figure 4 MSM-weighted intracellular minus extracellular gating distance versus AtSWEET13 transmembrane channel Z position of the closest GLC molecule carbon atom to the Trp58-Trp180 binding pocket S-9

S. Figure 5 MSM-weighted intracellular minus extracellular gating distance versus AtSWEET13 transmembrane channel Z position of the closest GLC molecule oxygen atom to the Trp58-Trp180 binding pocket S-10

S. Figure 6 MSM-weighted intracellular minus extracellular gating distance versus AtSWEET13 transmembrane channel Z position of the closest SUC molecule fructosyl carbon atom to the Trp58-Trp180 binding pocket S-11

S. Figure 7 MSM-weighted intracellular minus extracellular gating distance versus AtSWEET13 transmembrane channel Z position of the closest SUC molecule fructosyl oxygen atom to the Trp58-Trp180 binding pocket S-12

S. Figure 8 MSM-weighted intracellular minus extracellular gating distance versus AtSWEET13 transmembrane channel Z position of the closest SUC molecule glucosyl carbon atom to the Trp58-Trp180 binding pocket S-13

S. Figure 9 MSM-weighted intracellular minus extracellular gating distance versus AtSWEET13 transmembrane channel Z position of the closest SUC molecule glucosyl oxygen atom to the Trp58-Trp180 binding pocket S-14

S. Figure 10 Unweighted feature-diverse tICA decomposition landscapes S-15

S. Figure 11 Similar AtSWEET13 pore radius aperture is maintained regardless of substrate transported S-16

S. Figure 12 MSM-weighted θ_{xy} analysis versus AtSWEET13 transmembrane channel Z position of the closest GLC molecule carbon atom to the Trp58-Trp180 binding pocket S-17

S. Figure 13 MSM-weighted θ_{xy} analysis versus AtSWEET13 transmembrane channel Z position of the closest GLC molecule oxygen atom to the Trp58-Trp180 binding pocket S-18

S. Figure 14 MSM-weighted θ_{xy} analysis versus AtSWEET13 transmembrane channel Z position of the closest SUC molecule glucosyl carbon atom to the Trp58-Trp180 binding pocket S-19

S. Figure 15 MSM-weighted θ_{xy} analysis versus AtSWEET13 transmembrane channel Z position of the closest SUC molecule glucosyl oxygen atom to the Trp58-Trp180 binding pocket S-20

S. Figure 16 MSM-weighted θ_{xy} analysis versus AtSWEET13 transmembrane channel Z position of the closest SUC molecule fructosyl carbon atom to the Trp58-Trp180 binding pocket S-21

S. Figure 17 MSM-weighted θ_{xy} analysis versus AtSWEET13 transmembrane channel Z position of the closest SUC molecule fructosyl oxygen atom to the Trp58-Trp180 binding pocket S-22

S. Figure 18 MSM-weighted θ_{xz} analysis versus AtSWEET13 transmembrane channel Z position of the closest GLC molecule carbon atom to the Trp58-Trp180 binding pocket S-23

S. Figure 19 MSM-weighted θ_{xz} analysis versus AtSWEET13 transmembrane channel Z position of the closest GLC molecule oxygen atom to the Trp58-Trp180 binding pocket S-24

S. Figure 20 MSM-weighted θ_{xz} analysis versus AtSWEET13 transmembrane channel Z position of the closest SUC molecule glucosyl carbon atom to the Trp58-Trp180 binding pocket S-25

S. Figure 21 MSM-weighted θ_{xz} analysis versus AtSWEET13 transmembrane channel Z position of the closest SUC molecule glucosyl oxygen atom to the Trp58-Trp180 binding pocket S-26

S. Figure 22 MSM-weighted θ_{xz} analysis versus AtSWEET13 transmembrane channel Z position of the closest SUC molecule fructosyl carbon atom to the Trp58-Trp180 binding pocket S-27

S. Figure 23 MSM-weighted θ_{xz} analysis versus AtSWEET13 transmembrane channel Z position of the closest SUC molecule fructosyl oxygen atom to the Trp58-Trp180 binding pocket S-28

S. Figure 24 MSM-weighted θ_{yz} analysis versus AtSWEET13 transmembrane channel Z position of the closest GLC molecule carbon atom to the Trp58-Trp180 binding pocket S-29

S. Figure 25 MSM-weighted θ_{yz} analysis versus AtSWEET13 transmembrane channel Z position of the closest GLC molecule oxygen atom to the Trp58-Trp180 binding pocket S-30

S. Figure 26 MSM-weighted θ_{yz} analysis versus AtSWEET13 transmembrane channel Z position of the closest SUC molecule glucosyl carbon atom to the Trp58-Trp180 binding pocket S-31

S. Figure 27 MSM-weighted θ_{yz} analysis versus AtSWEET13 transmembrane channel Z position of the closest SUC molecule glucosyl oxygen atom to the Trp58-Trp180 binding pocket S-32

S. Figure 28 MSM-weighted θ_{yz} analysis versus AtSWEET13 transmembrane channel Z position of the closest SUC molecule fructosyl carbon atom to the Trp58-Trp180 binding pocket S-33

S. Figure 29 MSM-weighted θ_{yz} analysis versus AtSWEET13 transmembrane channel Z position of the closest SUC molecule fructosyl oxygen atom to the Trp58-Trp180 binding pocket S-34

S. Figure 30 GLC and SUC structures with highlighted functional considered critical for molecular recognition by AtSWEET13 S-35

S. Figure 31 Discriminative sugar transport events for AtSWEET13 S-36

S. Figure 32 GetContacts analysis for representative frames of metastable states throughout GLC transport S-37

S. Figure 33 GetContacts analysis for representative frames of metastable states throughout SUC transport S-38

S. Figure 34 C-H···π stacking and other interactions between GLC and AtSWEET13 throughout molecular recognition and transport S-39

S. Figure 35 C-H···π stacking and other interactions between SUC and AtSWEET13 throughout molecular recognition and transport S-40

S. Figure 36 Raw counts versus MSM population in each clustered state S-41

S. Figure 37 Chapman-Kolmogorov test for the MSM of *apo* AtSWEET13 conformational dynamics and transport S-42

S. Figure 38 Chapman-Kolmogorov test for the MSM of AtSWEET13 conformational dynamics during GLC transport S-43

S. Figure 39 Chapman-Kolmogorov test for the MSM of AtSWEET13 conformational dynamics during SUC transport S-44

S. Figure 40 Implied timescale plots calculated with Bayesian error S-45

S. Figure 41 MSM-weighted bootstrap error plots for adaptive sampling of gating landscapes S-46

S. Figure 42 MSM-weighted bootstrap error plots for adaptive sampling of intracellular minus extracellulargating distance versus AtSWEET13 transmembrane channel ligand Z position landscapes S-47

S. Figure 43 MSM-weighted bootstrap error plots for adaptive sampling of $\boldsymbol{\theta}$ rotation analyses presented in Main Text Figure 4 S-48

S. Table 1 Finalized features used for MSM discretization S-49

S. Table 2 Descriptor correlation to tIC1 from feature-diverse tICA decomposition of GLC transport S-50

S. Table 3 Descriptor correlation to tIC1 from feature-diverse tICA decomposition of SUC transport S-51

S. Table 4 Descriptor correlation to tIC1 from feature-diverse tICA decomposition of *apo* transport S-52

S. Table 5 Descriptor correlation to tIC2 from feature-diverse tICA decomposition of SUC transport S-53

S. Table 6 Descriptor correlation to tIC2 from feature-diverse tICA decomposition of GLC transport S-54

S. Table 7 Realistic bilayer composition used during AtSWEET13 simulations S-55

S. Table 8 Finalized MSM hyperparameters S-56

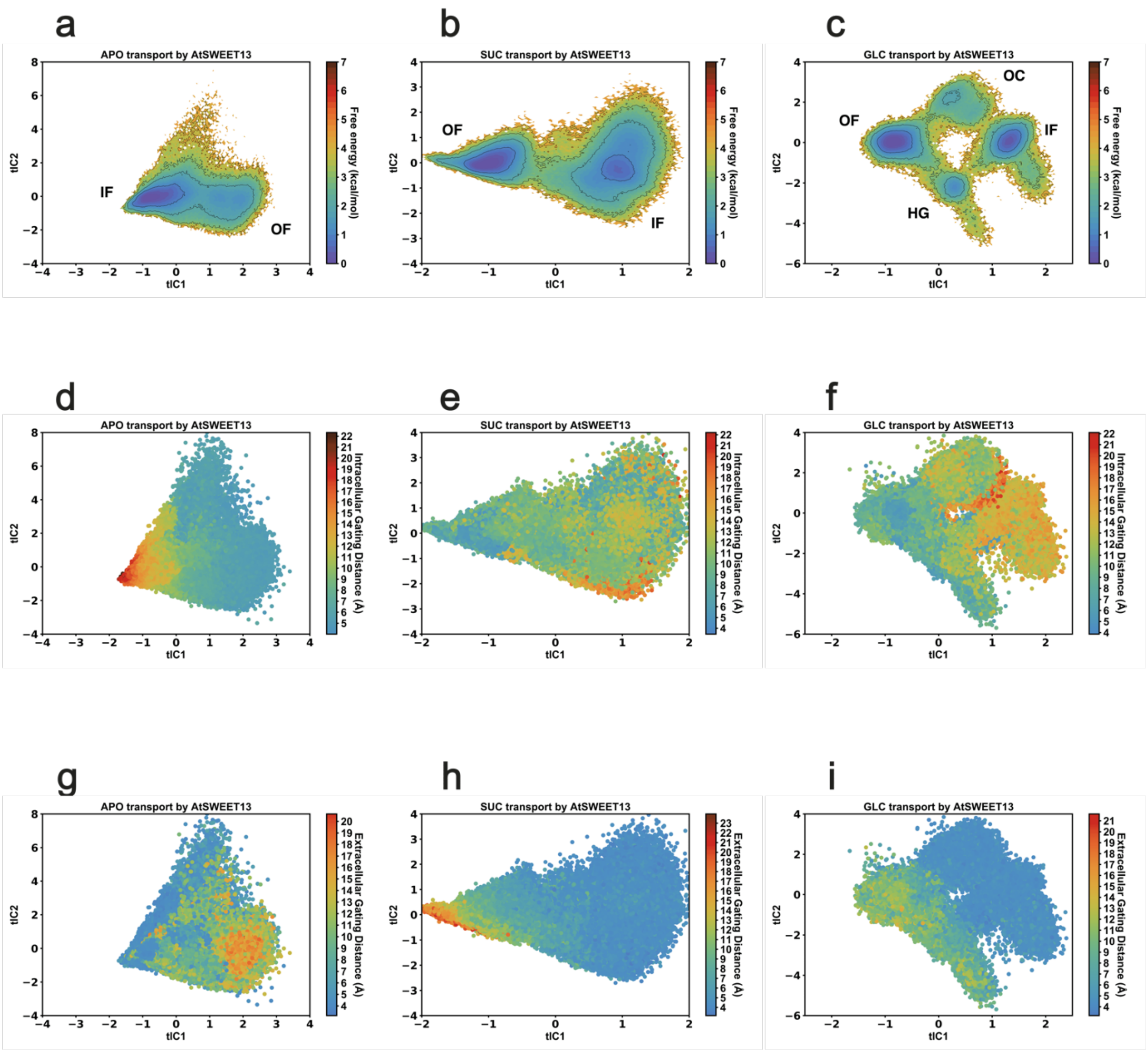

Supplementary Figure 1. tICA decomposition given simplified MSM features for AtSWEET13 transport processes. (**a**) *Apo*, (**b**) SUC, and (**c**) GLC MSM-weighted tICA transport landscapes. tICA decomposition represented as scatterplots are colored by extent of intracellular gating distance for (**d**) *Apo*, (**e**) SUC, and (**f**) GLC transport processes. tICA decomposition represented as scatterplots are colored by extent of extracellular gating distance for (**g**) Apo, (**h**) SUC, and (**i**) GLC transport processes.

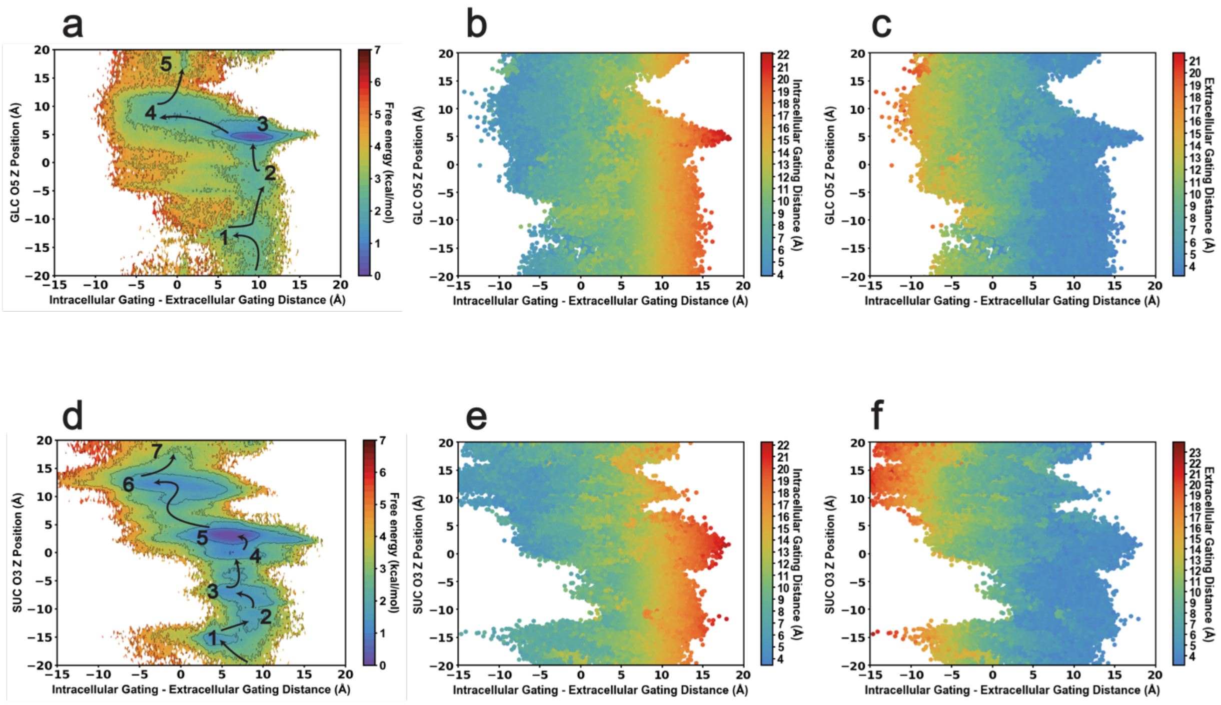

Supplementary Figure 2. Scatterplot representation of transport versus the difference in gating highlights that substrate transport proceeds through an hour-glass state. GLC transport versus the difference in intracellular versus extracellular gating represented using (**a**) an MSM-weighted free energy landscape, (**b**) a scatterplot colored by extent of intracellular gating, and (**c**) a scatterplot colored by extent of extracellular gating. SUC transport versus the difference in intracellular versus extracellular gating represented using (**d**) an MSM-weighted free energy landscape, (**e**) a scatterplot colored by extent of intracellular gating, and (**f**) a scatterplot colored by extent of extracellular gating.

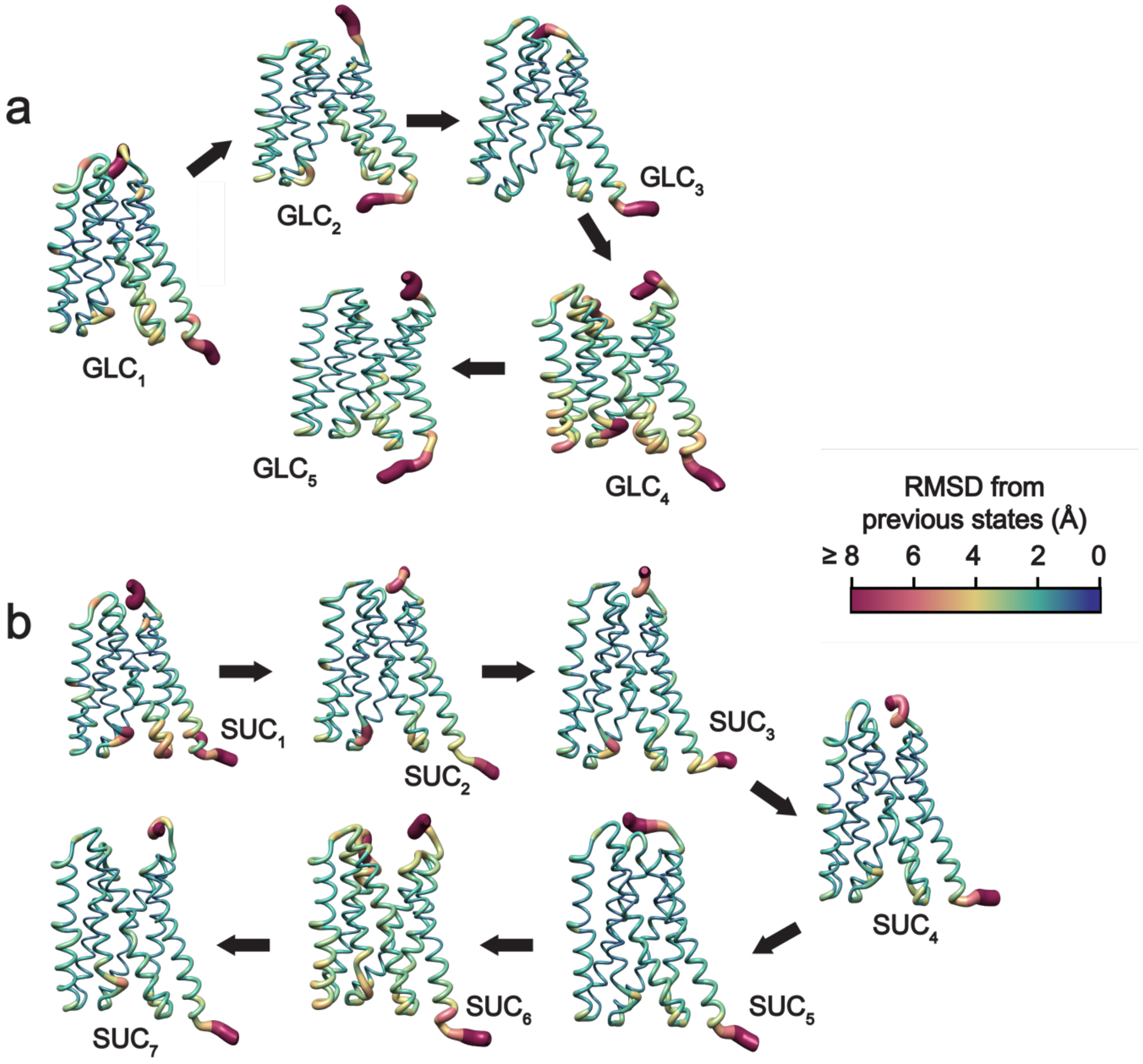

Supplementary Figure 3. AtSWEET13 transport cycles for GLC and SUC translocation implicate minimal conformational change outside of commitment to alternate access. (**a**) Protein snapshots taken for different conformational states depicting GLC transport, where worms visualization represents protein residue RMSD values in Å units. (**b**) Protein snapshots taken for different conformational states depicting SUC transport using similar worms visualization as seen in (**a**). RMSDs are calculated as an average in comparison to the number of states found at the center of each energetic minima characterizing each enumerated state in Main Text Figure 2. RMSDs shown for states GLC_1 and SUC_1 are in comparison to the 5XPD crystal structure conformation.

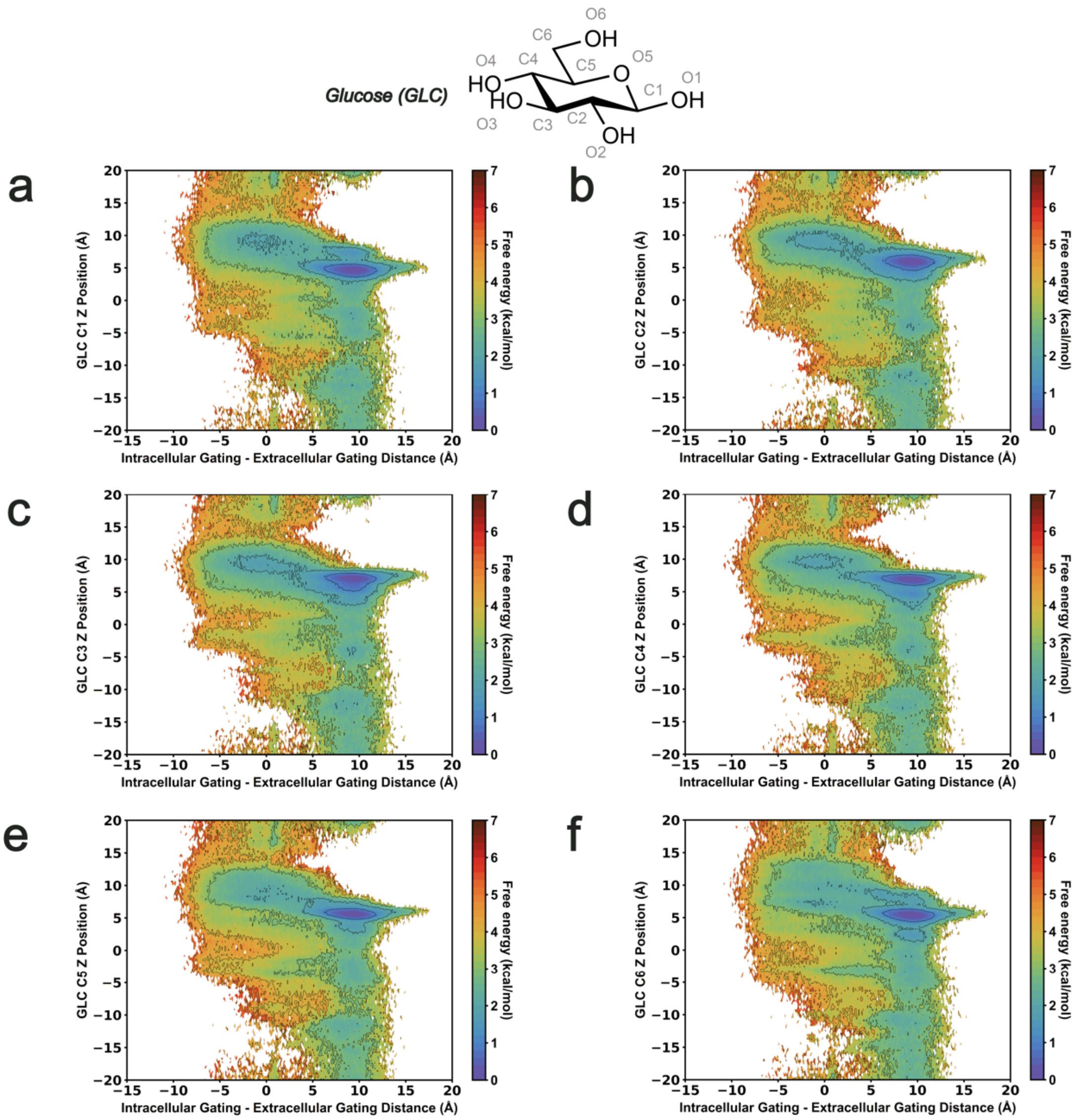

Supplementary Figure 4. MSM-weighted intracellular minus extracellular gating distance versus AtSWEET13 transmembrane channel Z position of the closest GLC molecule carbon atom to the Trp58-Trp180 binding pocket. (**a**) GLC C1. (**b**) GLC C2. (**c**) GLC C3. (**d**) GLC C4. (**e**) GLC C5. (**f**) GLC C6.

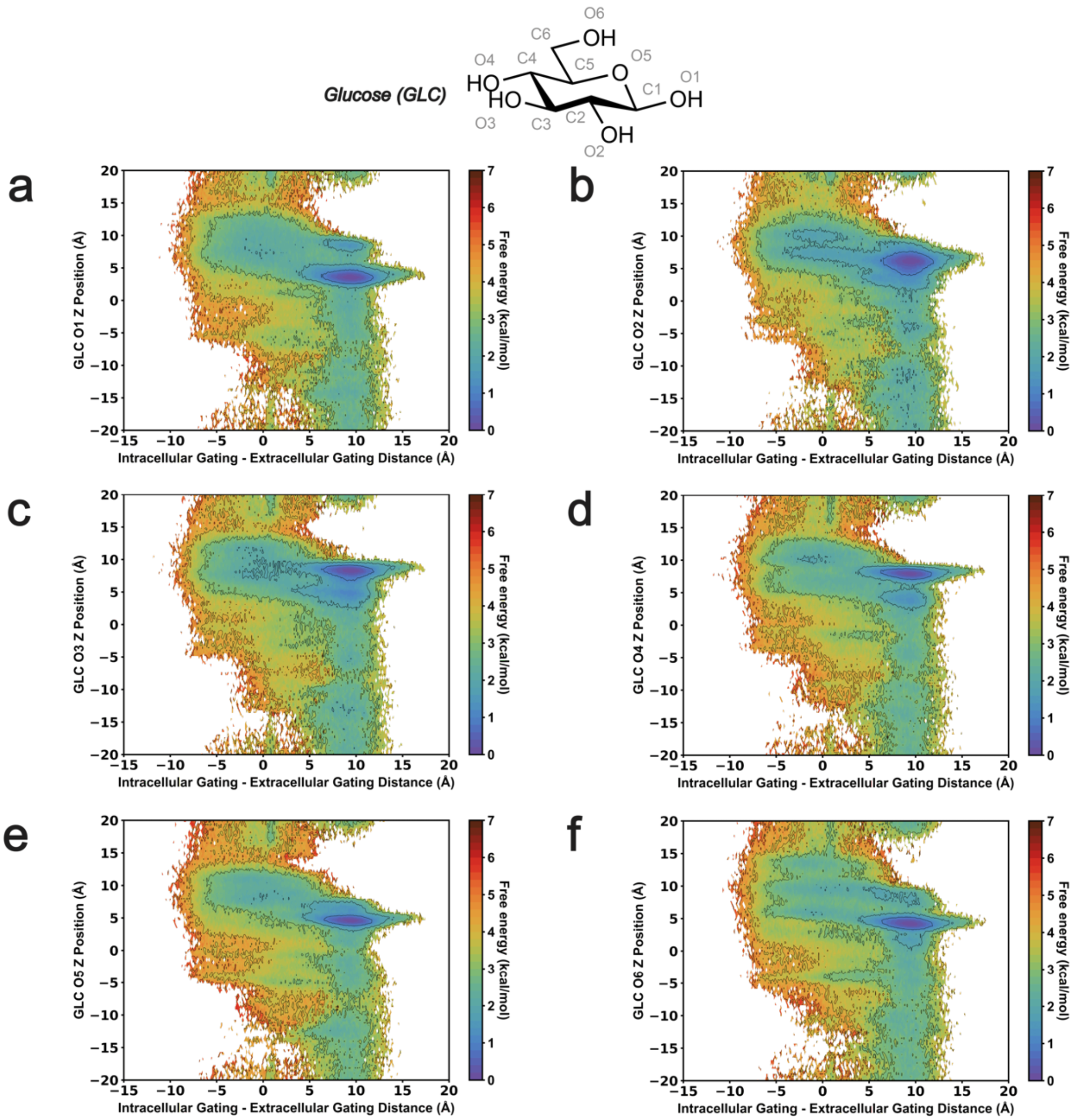

Supplementary Figure 5. MSM-weighted intracellular minus extracellular gating distance versus AtSWEET13 transmembrane channel Z position of the closest GLC molecule oxygen atom to the Trp58-Trp180 binding pocket. (**a**) GLC O1. (**b**) GLC O2. (**c**) GLC O3. (**d**) GLC O4. (**e**) GLC O5. (**f**) GLC O6.

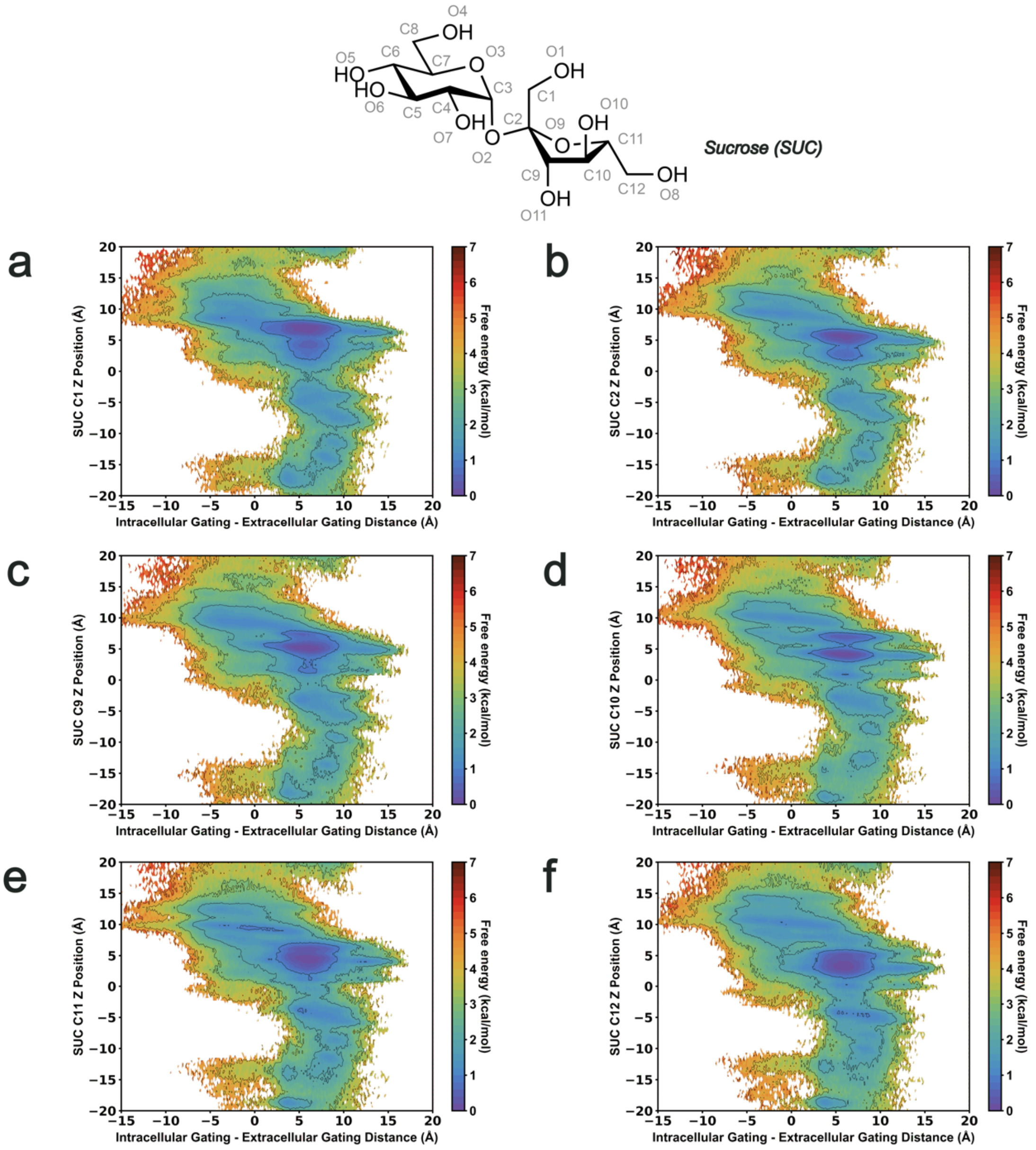

Supplementary Figure 6. MSM-weighted intracellular minus extracellular gating distance versus AtSWEET13 transmembrane channel Z position of the closest SUC molecule fructosyl carbon atom to the Trp58-Trp180 binding pocket. (**a**) SUC C1. (**b**) SUC C2. (**c**) SUC C9. (**d**) SUC C10. (**e**) SUC C11. (**f**) SUC C12.

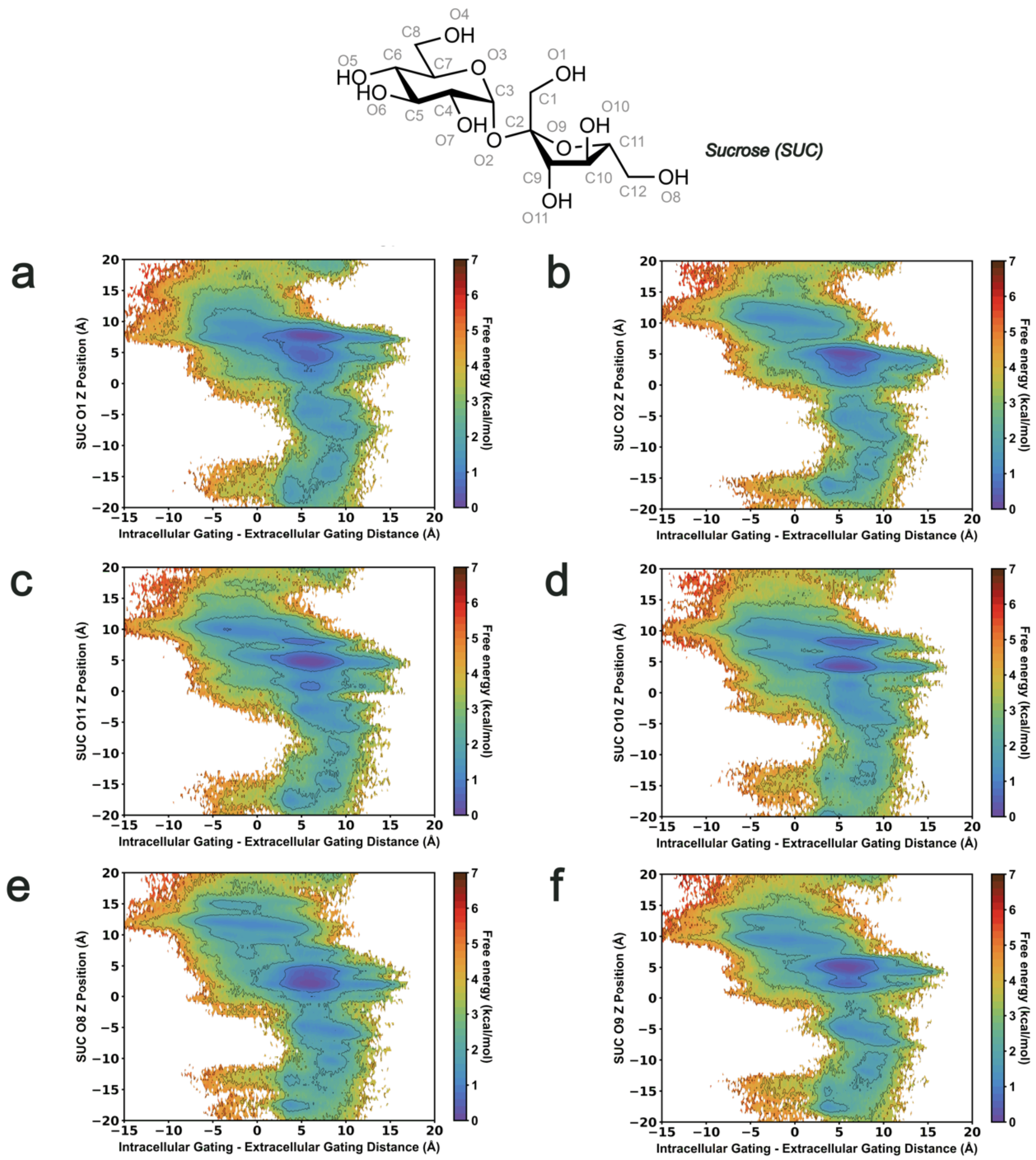

Supplementary Figure 7. MSM-weighted intracellular minus extracellular gating distance versus AtSWEET13 transmembrane channel Z position of the closest SUC molecule fructosyl oxygen atom to the Trp58-Trp180 binding pocket. (**a**) SUC O1. (**b**) SUC O2. (**c**) SUC O11. (**d**) SUC O10. (**e**) SUC O8. (**f**) SUC O9.

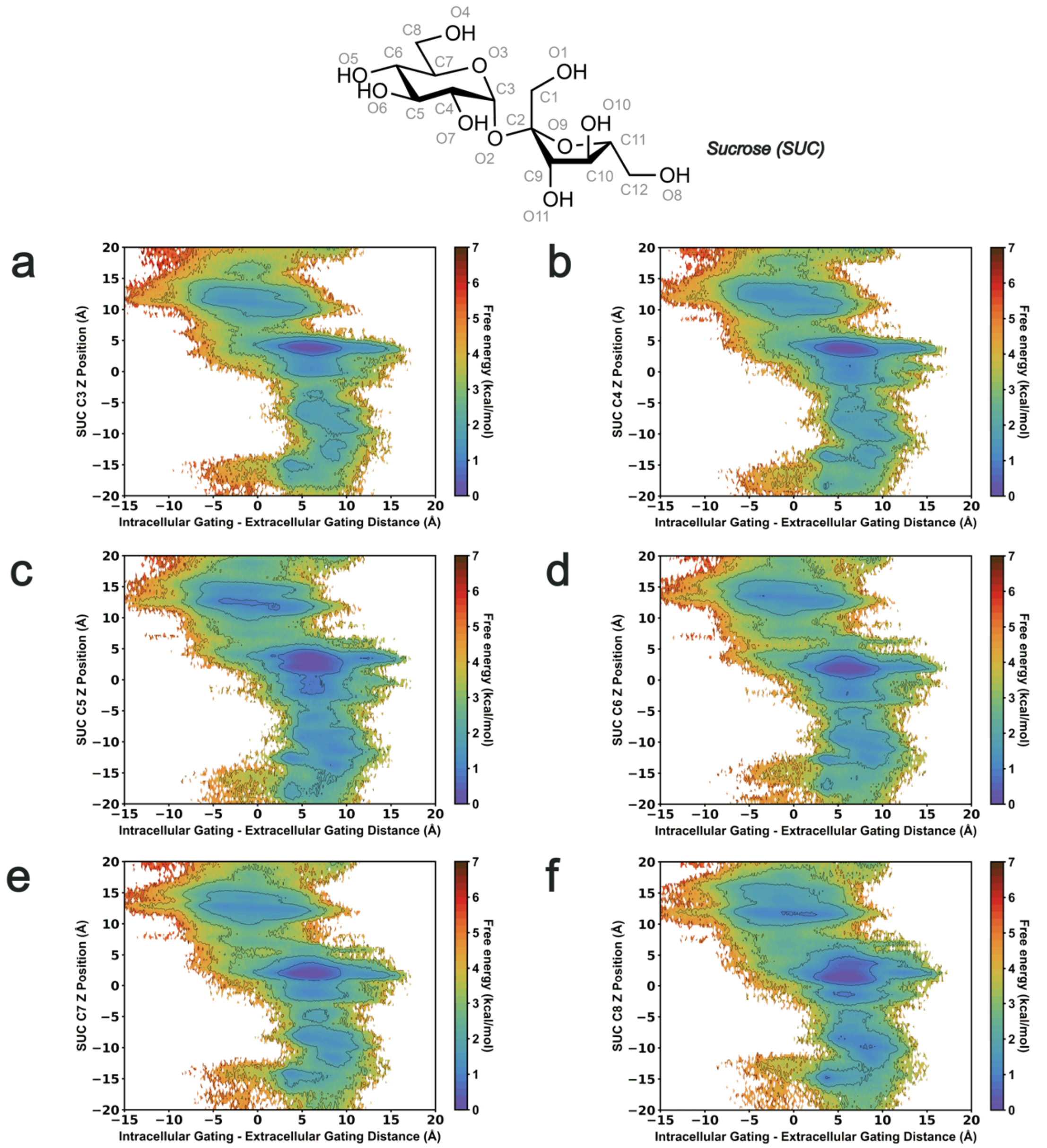

Supplementary Figure 8. MSM-weighted intracellular minus extracellular gating distance versus AtSWEET13 transmembrane channel Z position of the closest SUC molecule glucosyl carbon atom to the Trp58-Trp180 binding pocket. (**a**) SUC C3. (**b**) SUC C4. (**c**) SUC C5. (**d**) SUC C6. (**e**) SUC C7. (**f**) SUC C8.

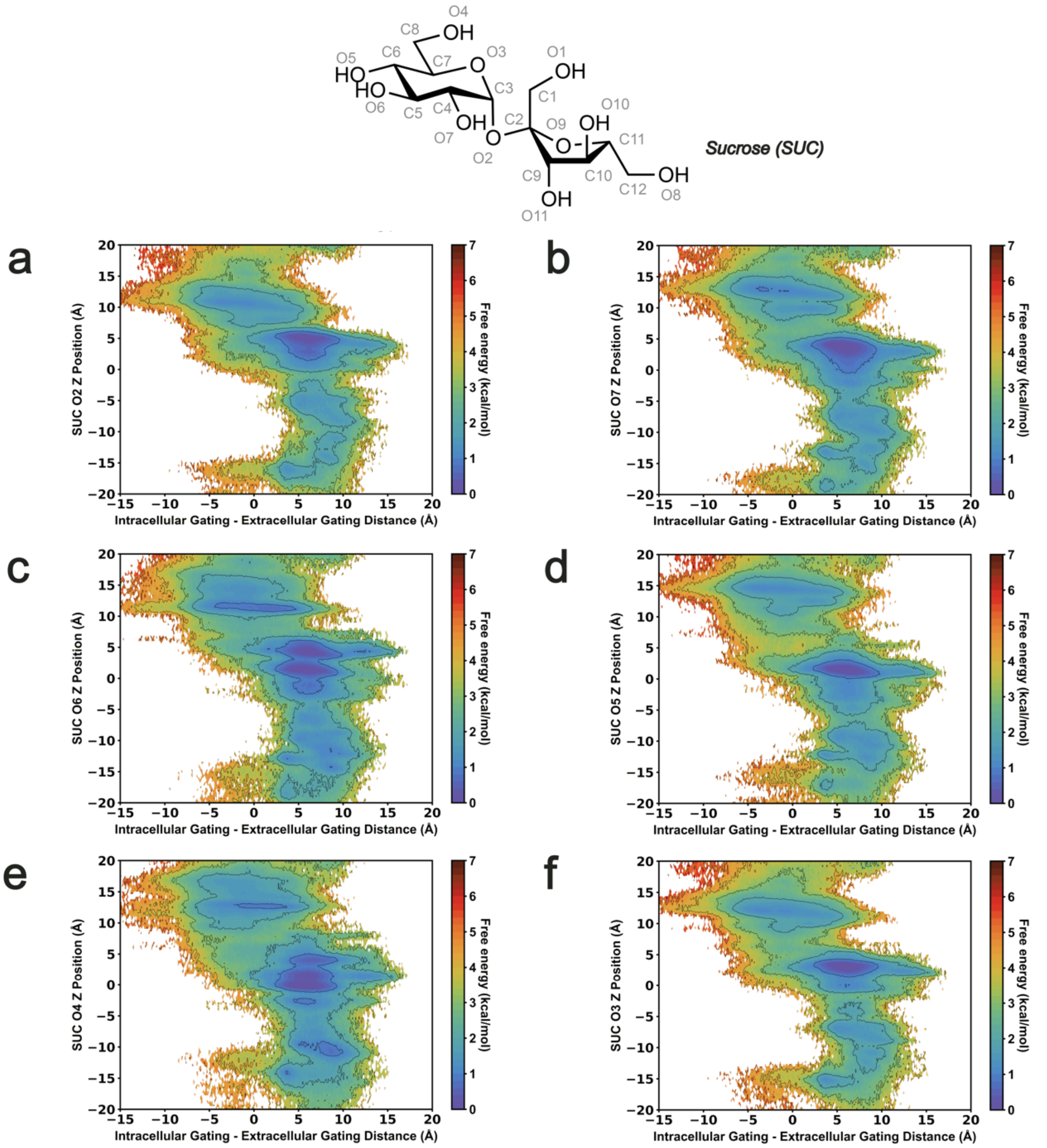

Supplementary Figure 9. MSM-weighted intracellular minus extracellular gating distance versus AtSWEET13 transmembrane channel Z position of the closest SUC molecule glucosyl oxygen atom to the Trp58-Trp180 binding pocket. (**a**) SUC O2. (**b**) SUC O7. (**c**) SUC O6. (**d**) SUC O5. (**e**) SUC O4. (**f**) SUC O3.

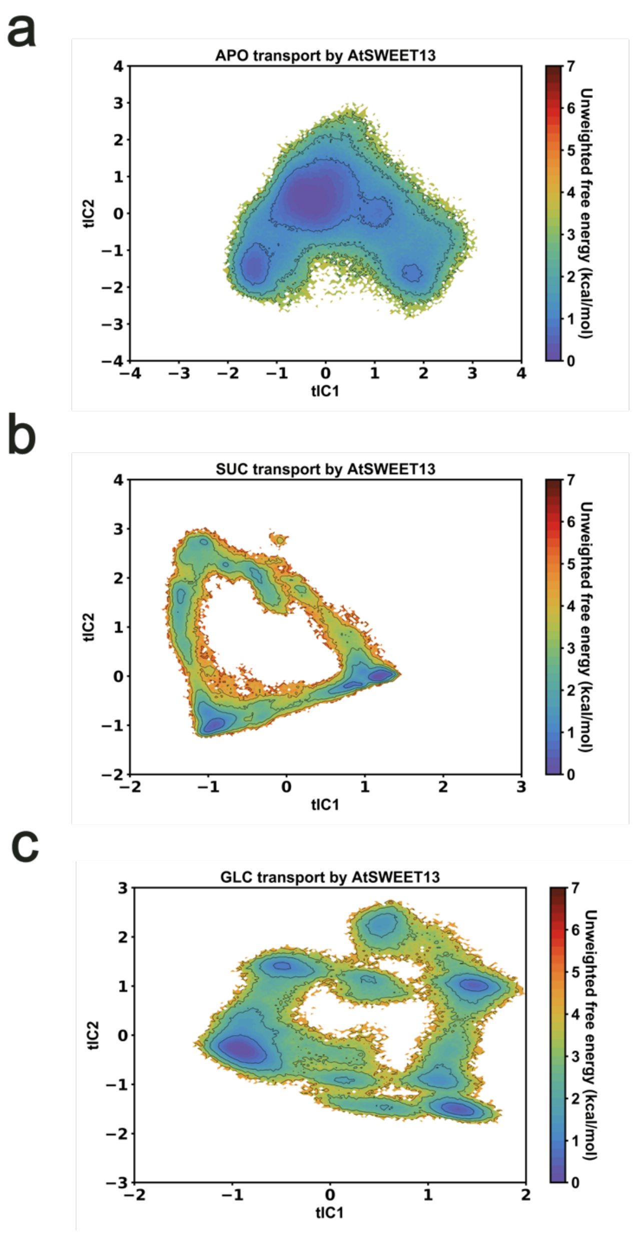

Supplementary Figure 10. Unweighted feature-diverse tICA decomposition landscapes for (**a**) *apo*, (**b**) SUC, and (**c**) GLC transport by AtSWEET13.

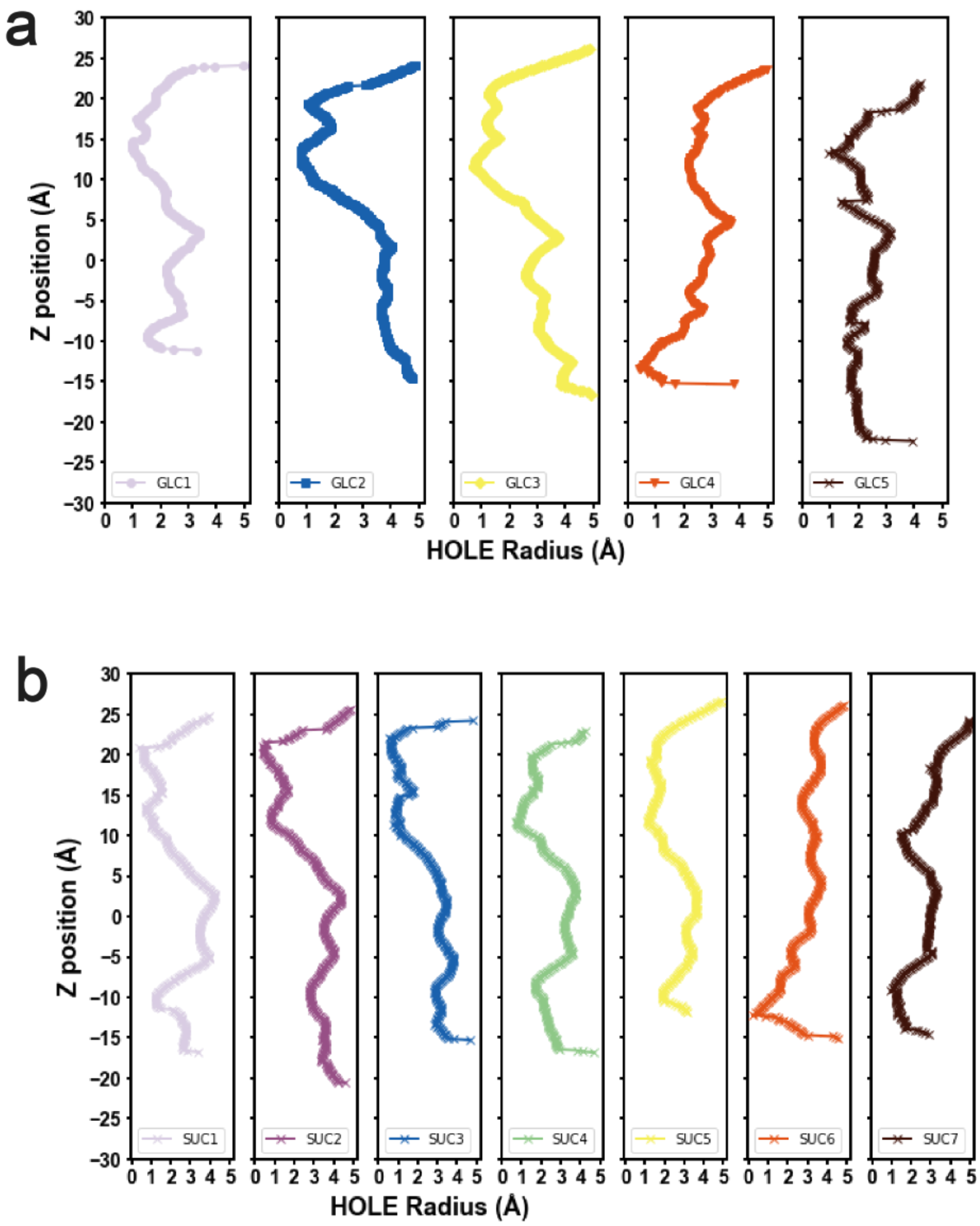

Supplementary Figure 11. Similar AtSWEET13 pore radius aperture is maintained regardless of substrate transported. (**a**) HOLE calculations of metastable states observed for GLC transport. (**b**) HOLE calculations of metastable states observed for SUC transport. State numbering is identical to states shown in Main Text Figure 2. GLC HOLE calculations shown here in Panel A correspond to the state numbering shown in Main Text Figure 2a and Supplementary Figure 3a. SUC HOLE calculations shown here in (**b**) correspond to the state numbering shown in Main Text Figure 2b and Supplementary Figure 3b.

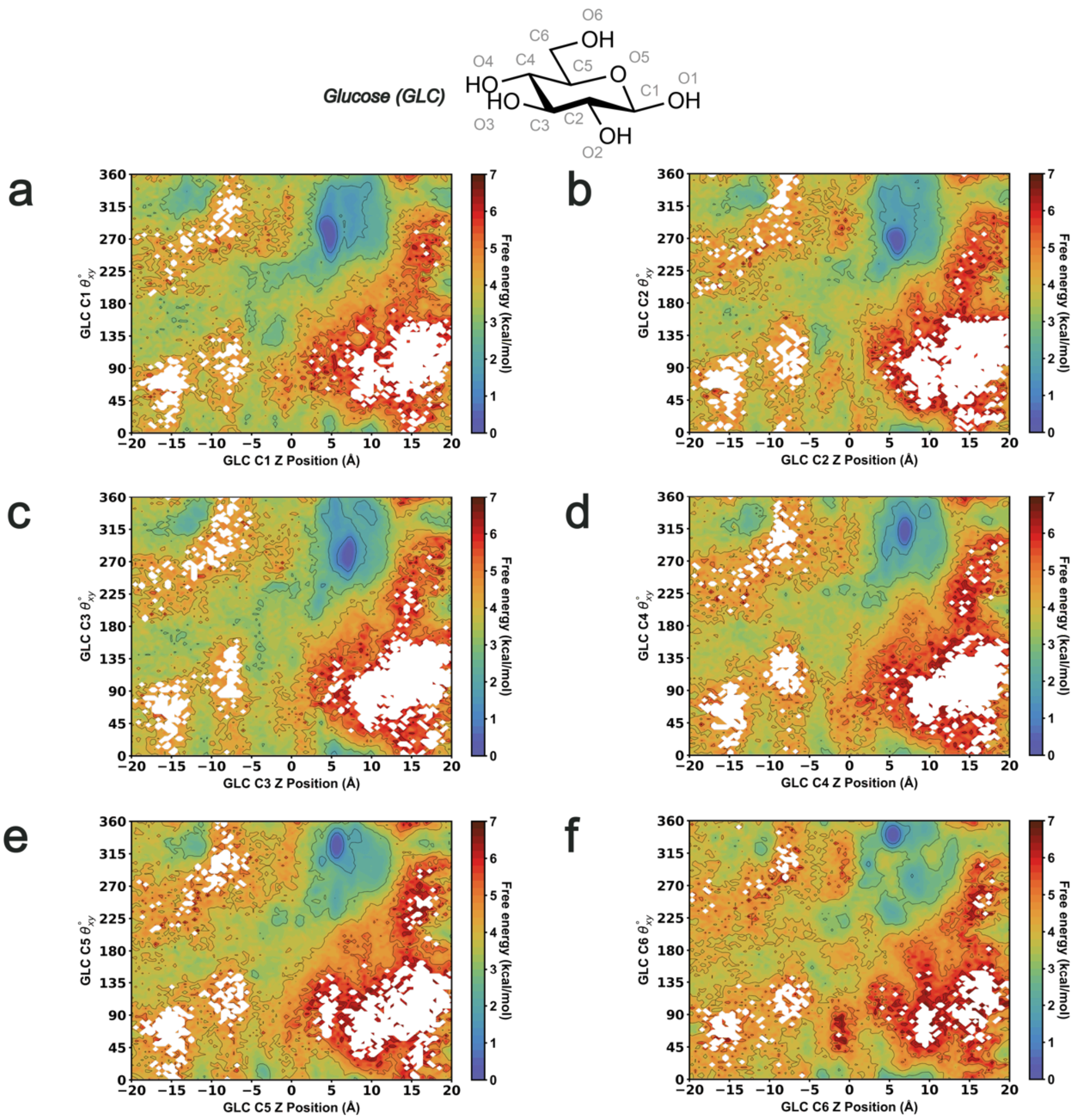

Supplementary Figure 12. MSM-weighted θ_{xy} analysis versus AtSWEET13 transmembrane channel Z position of the closest GLC molecule carbon atom to the Trp58-Trp180 binding pocket. (**a**) GLC C1. (**b**) GLC C2. (**c**) GLC C3. (**d**) GLC C4. (**e**) GLC C5. (**f**) GLC C6.

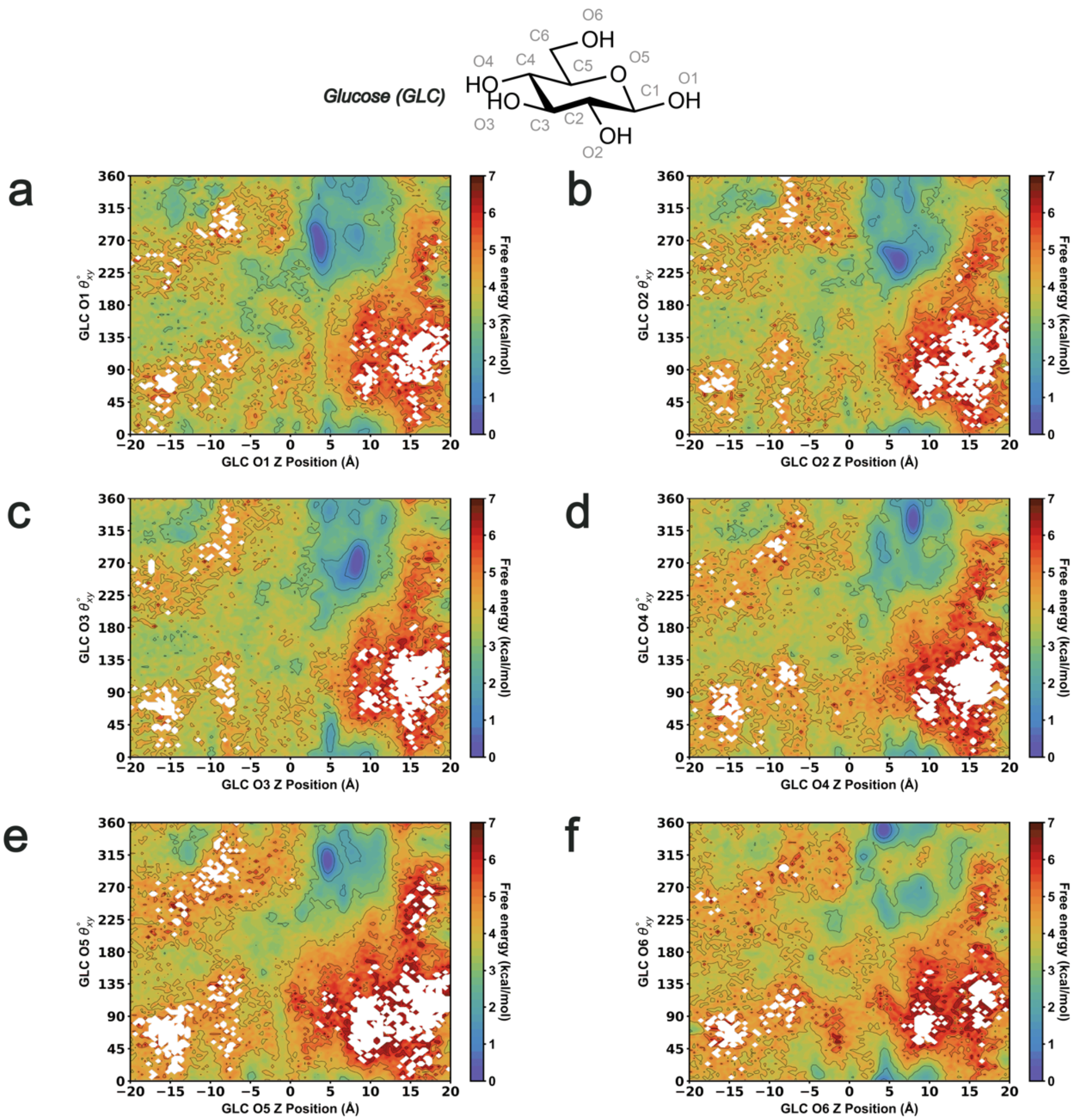

Supplementary Figure 13. MSM-weighted θ_{xy} analysis versus AtSWEET13 transmembrane channel Z position of the closest GLC molecule oxygen atom to the Trp58-Trp180 binding pocket. (**a**) GLC O1. (**b**) GLC O2. (**c**) GLC O3. (**d**) GLC O4. (**e**) GLC O5. (**f**) GLC O6.

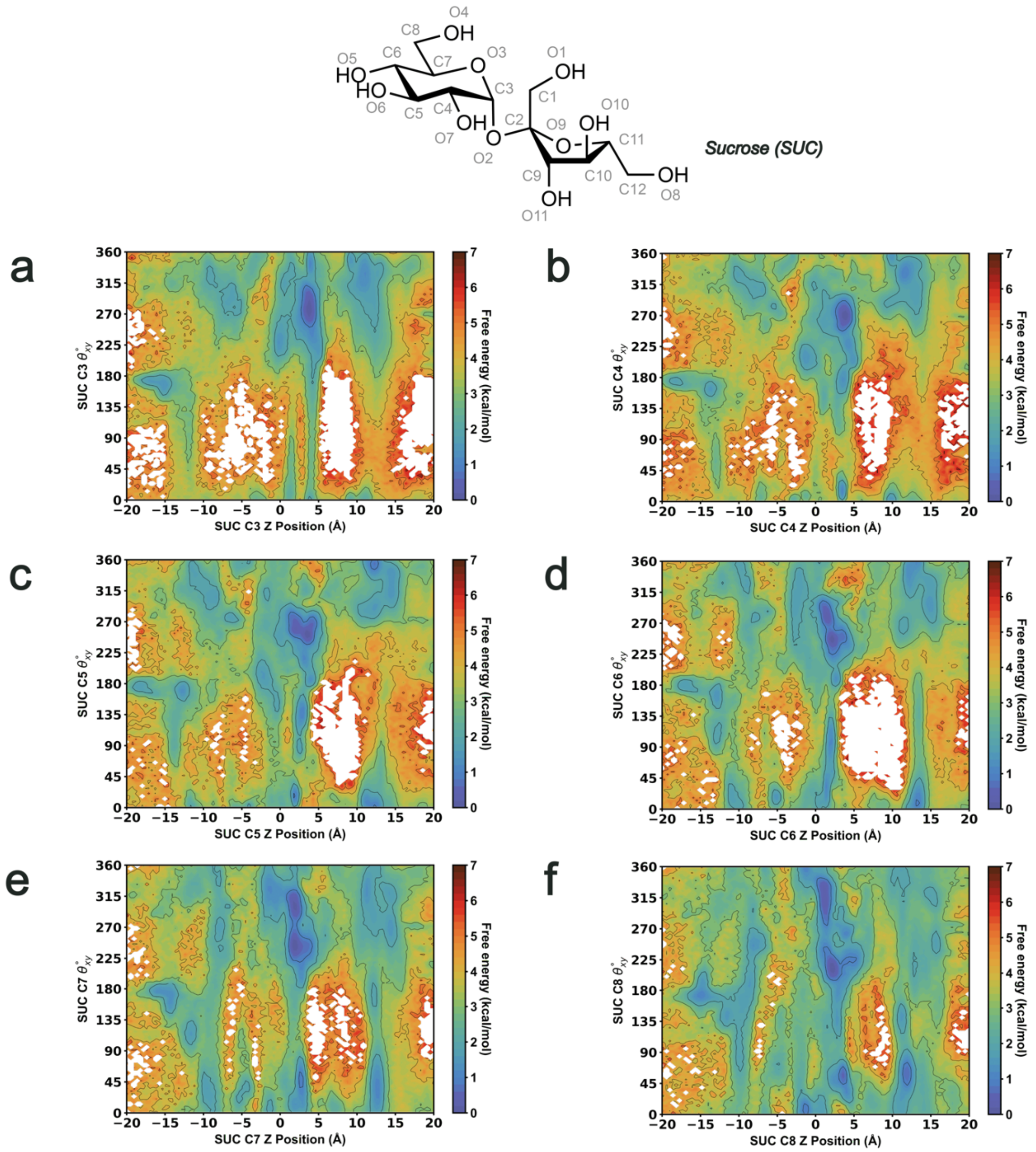

Supplementary Figure 14. MSM-weighted θ_{xy} analysis versus AtSWEET13 transmembrane channel Z position of the closest SUC molecule glucosyl carbon atom to the Trp58-Trp180 binding pocket. (**a**) SUC C3. (**b**) SUC C4. (**c**) SUC C5. (**d**) SUC C6. (**e**) SUC C7. (**f**) SUC C8.

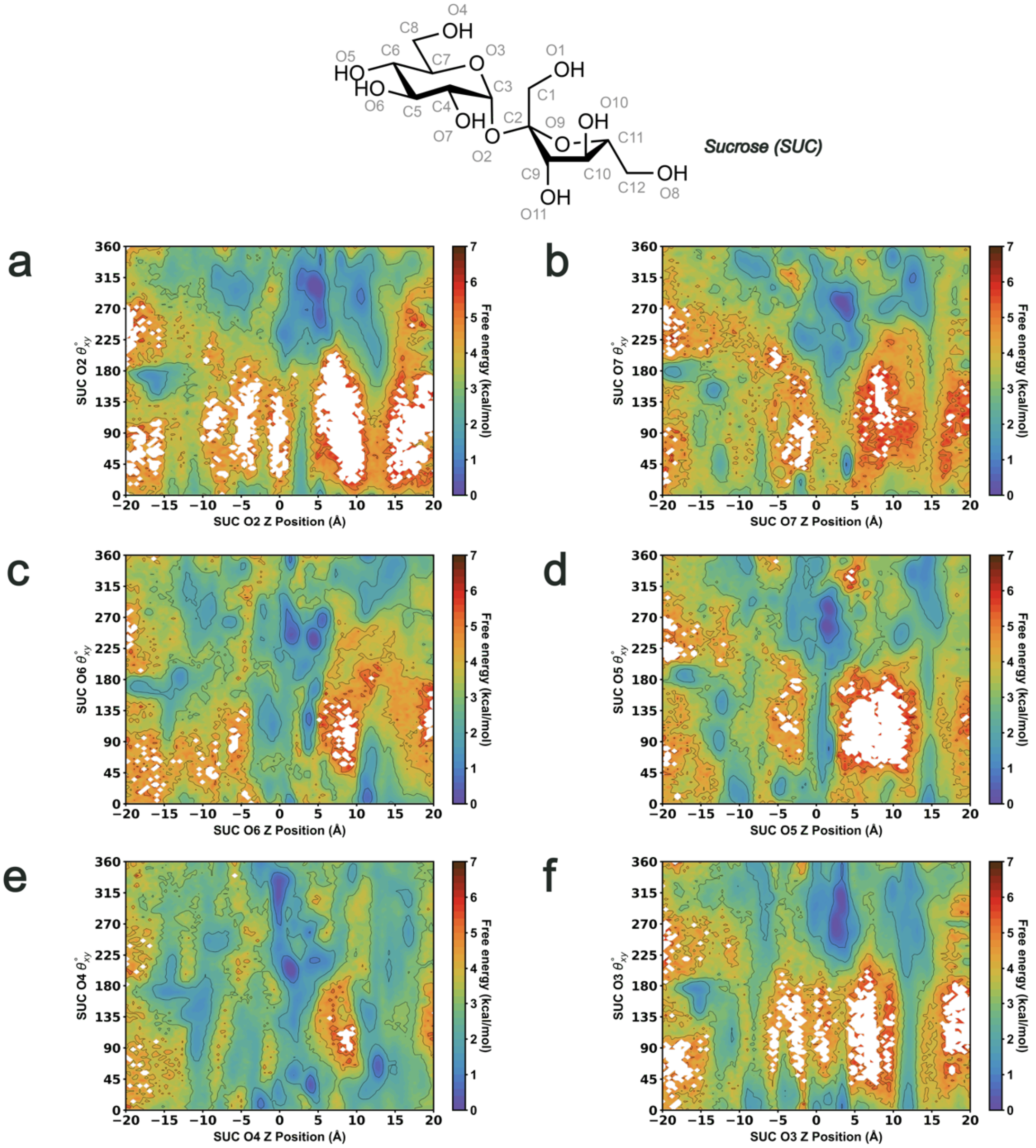

Supplementary Figure 15. MSM-weighted θ_{xy} analysis versus AtSWEET13 transmembrane channel Z position of the closest SUC molecule glucosyl oxygen atom to the Trp58-Trp180 binding pocket. (**a**) SUC O2. (**b**) SUC O7. (**c**) SUC O6. (**d**) SUC O5. (**e**) SUC O4. (**f**) SUC O3.

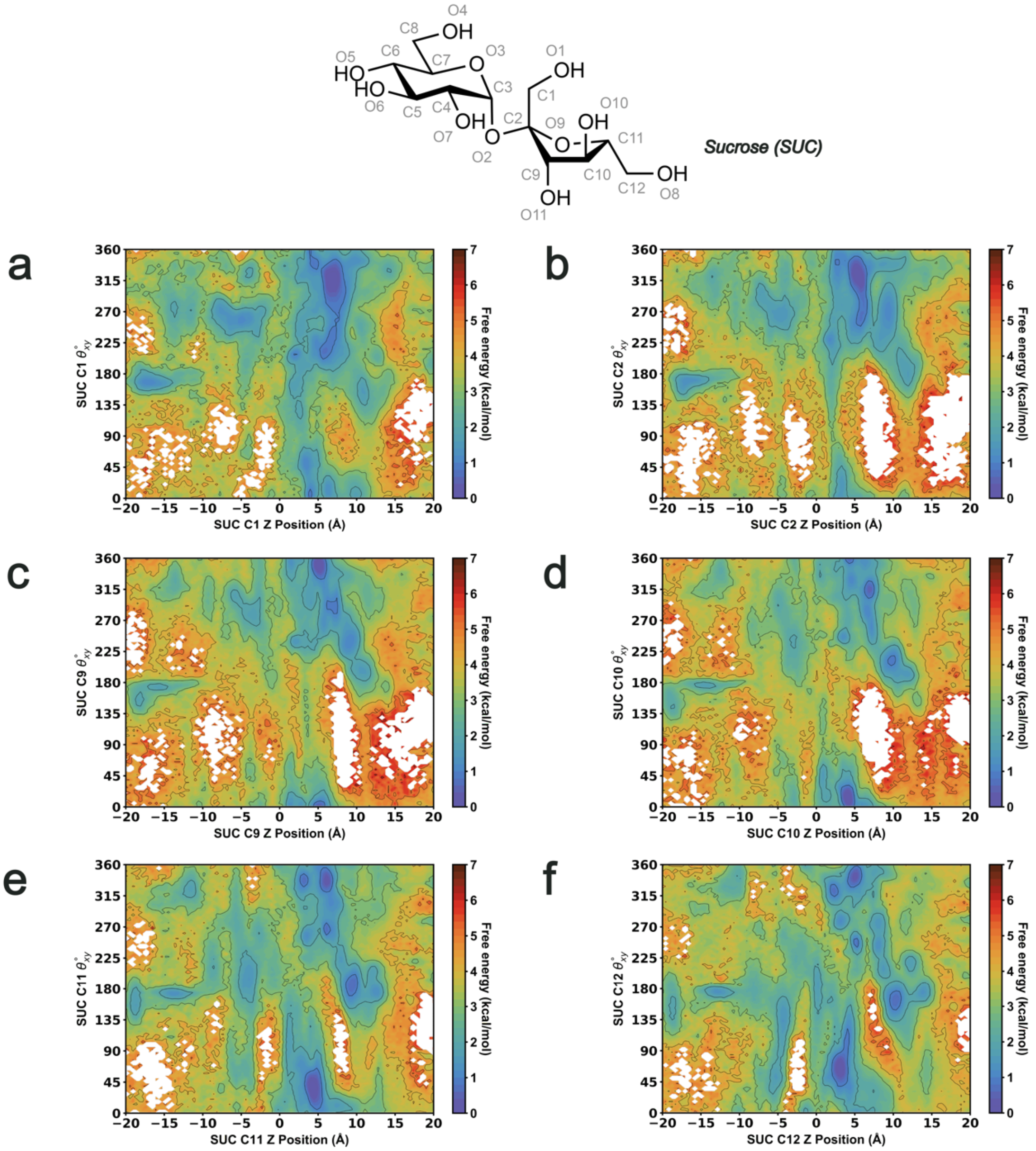

Supplementary Figure 16. MSM-weighted θ_{xy} analysis versus AtSWEET13 transmembrane channel Z position of the closest SUC molecule fructosyl carbon atom to the Trp58-Trp180 binding pocket. (**a**) SUC C1. (**b**) SUC C2. (**c**) SUC C9. (**d**) SUC C10. (**e**) SUC C11. (**f**) SUC C12.

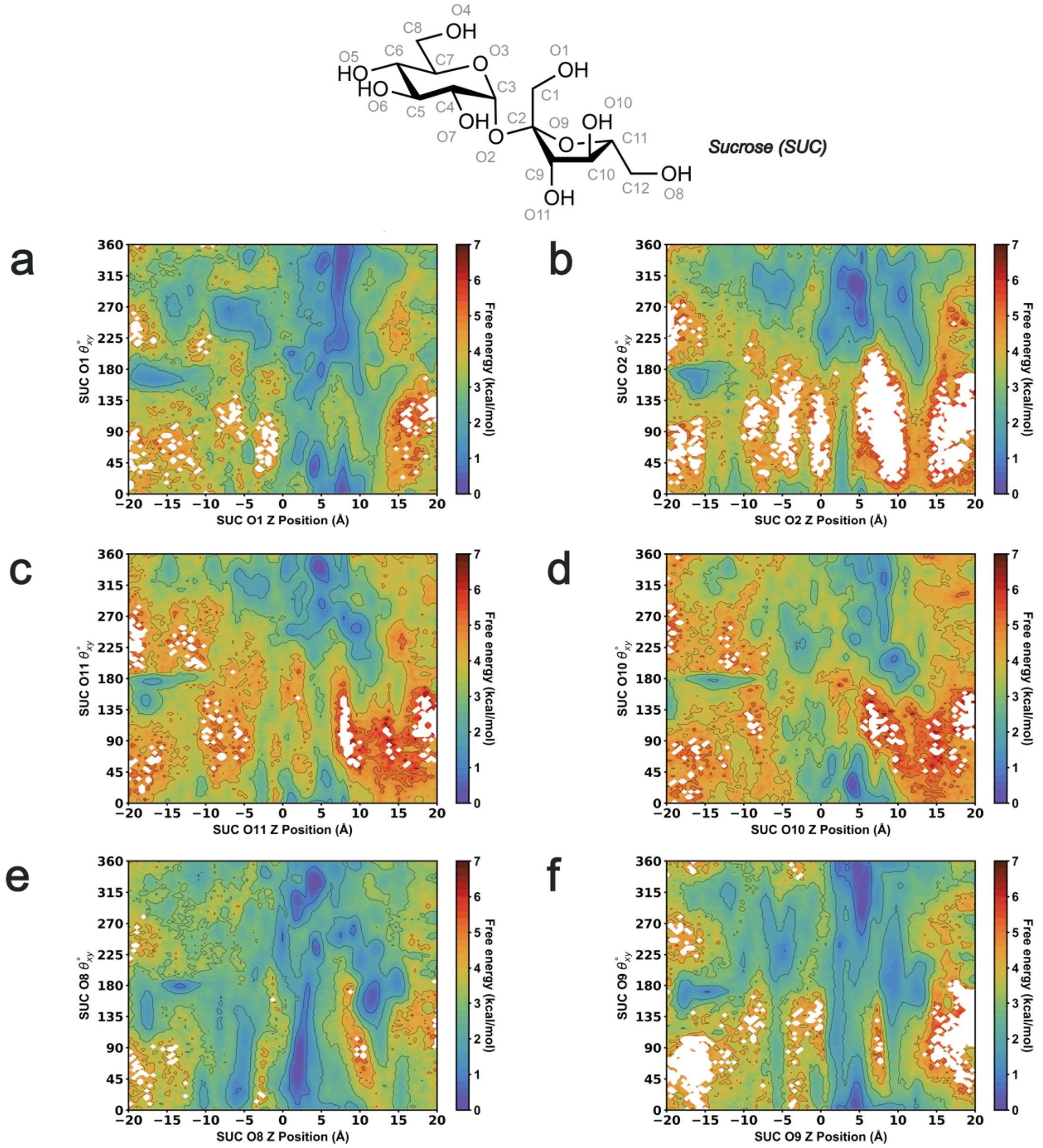

Supplementary Figure 17. MSM-weighted θ_{xy} analysis versus AtSWEET13 transmembrane channel Z position of the closest SUC molecule fructosyl oxygen atom to the Trp58-Trp180 binding pocket. (**a**) SUC O1. (**b**) SUC O2. (**c**) SUC O11. (**d**) SUC O10. (**e**) SUC O8. (**f**) SUC O9.

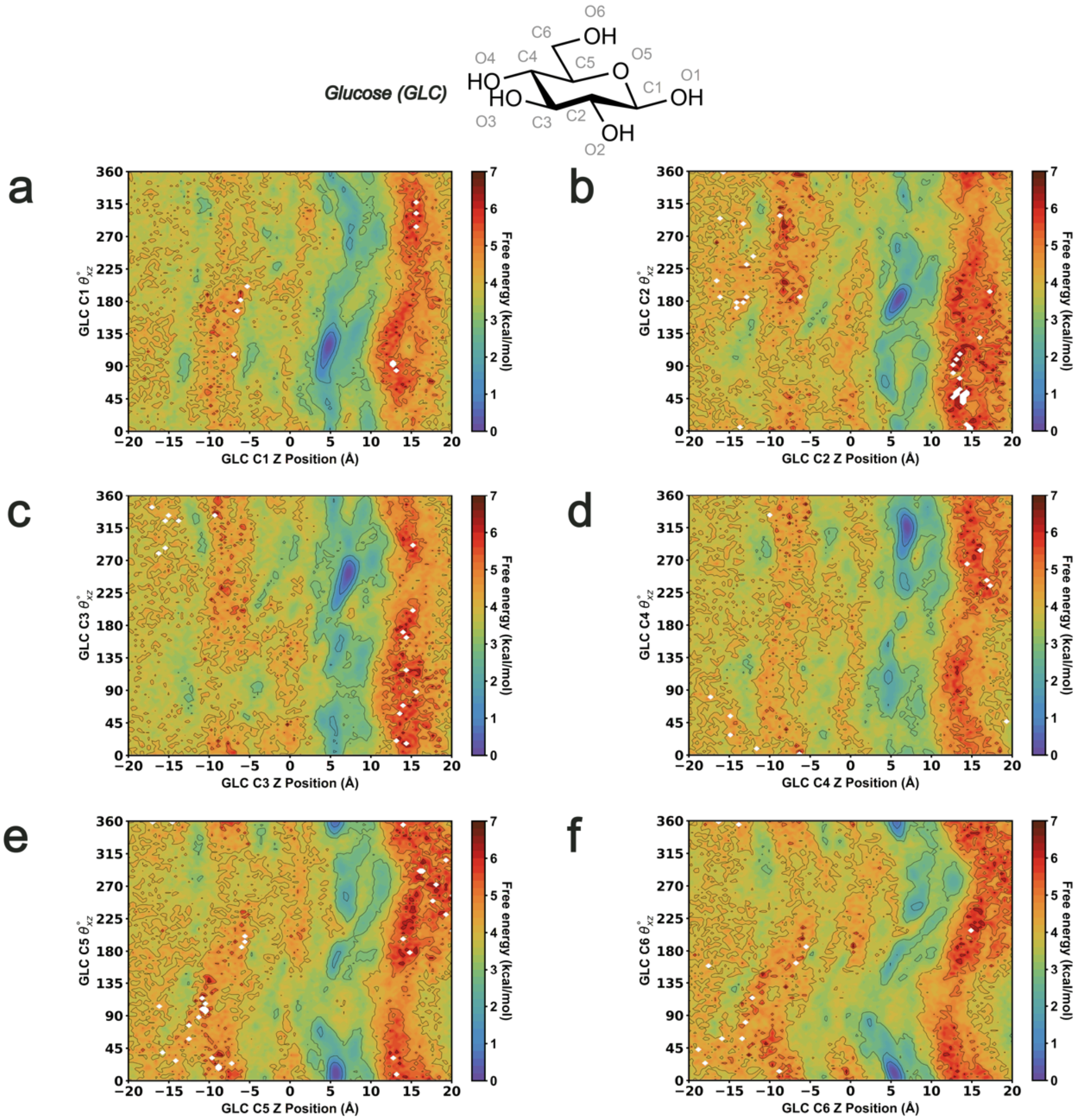

Supplementary Figure 18. MSM-weighted θ_{xz} analysis versus AtSWEET13 transmembrane channel Z position of the closest GLC molecule carbon atom to the Trp58-Trp180 binding pocket. (**a**) GLC C1. (**b**) GLC C2. (**c**) GLC C3. (**d**) GLC C4. (**e**) GLC C5. (**f**) GLC C6.

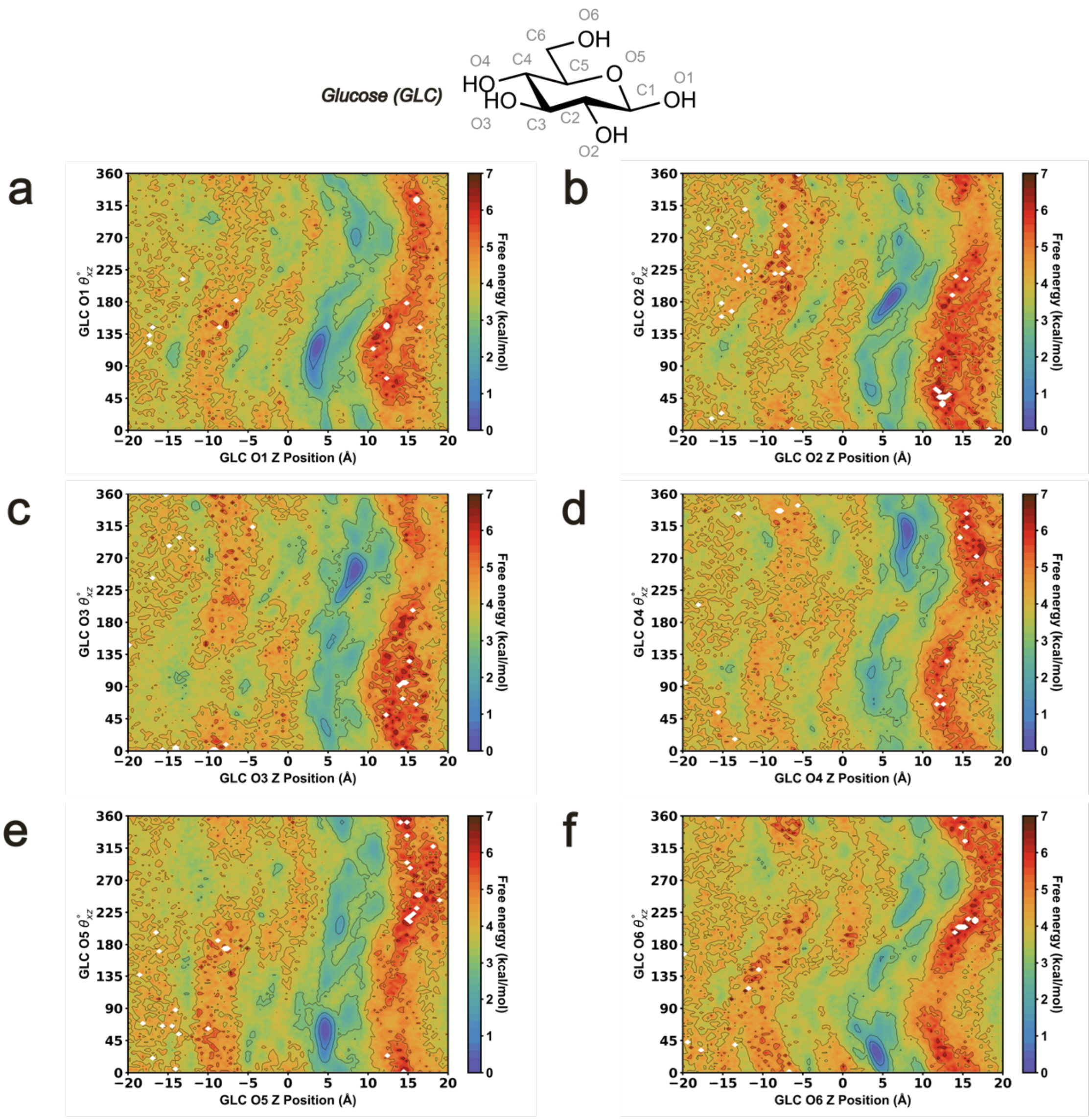

Supplementary Figure 19. MSM-weighted θ_{xz} analysis versus AtSWEET13 transmembrane channel Z position of the closest GLC molecule oxygen atom to the Trp58-Trp180 binding pocket. (**a**) GLC O1. (**b**) GLC O2. (**c**) GLC O3. (**d**) GLC O4. (**e**) GLC O5. (**f**) GLC O6.

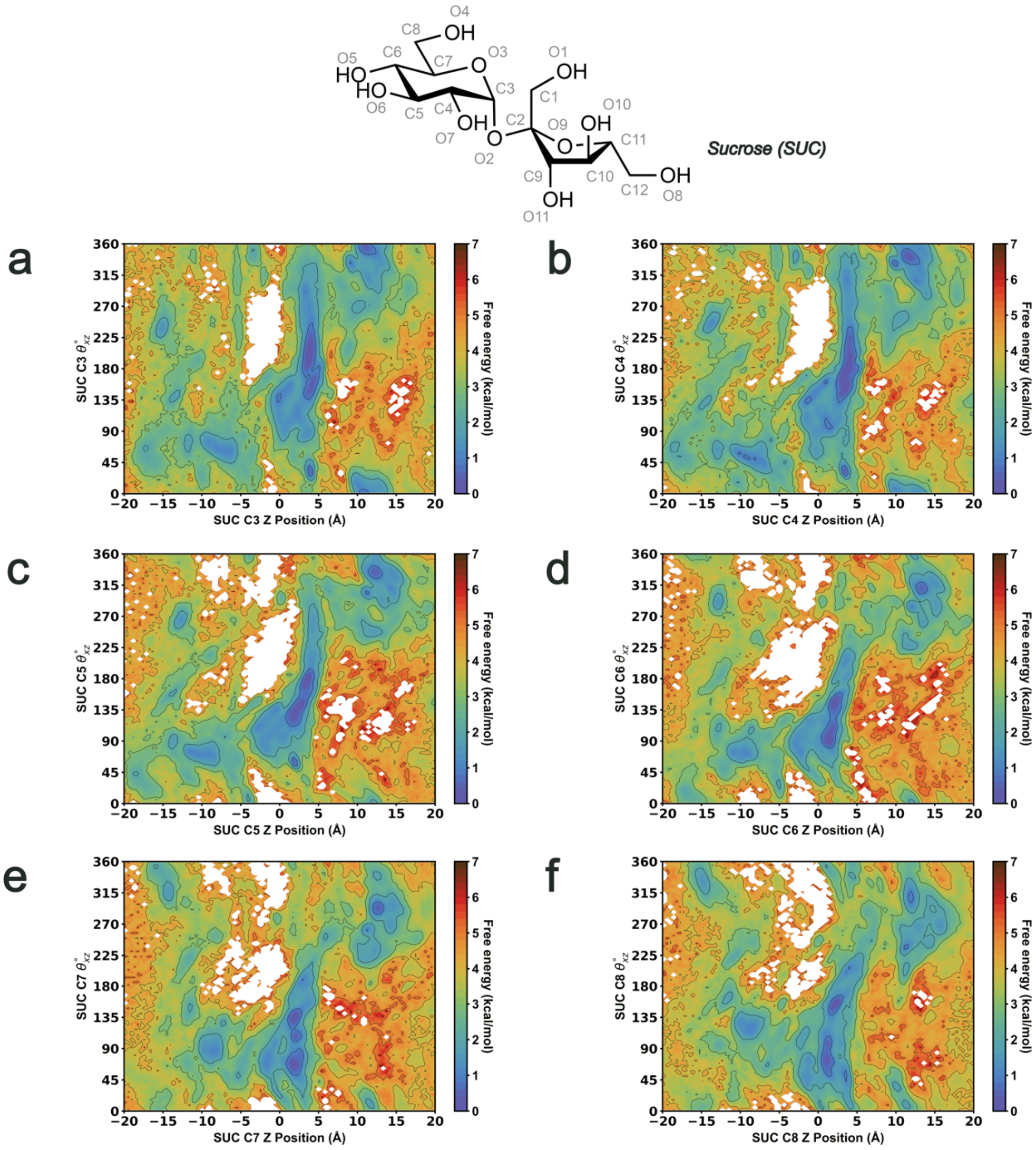

Supplementary Figure 20. MSM-weighted θ_{xz} analysis versus AtSWEET13 transmembrane channel Z position of the closest SUC molecule glucosyl carbon atom to the Trp58-Trp180 binding pocket. (**a**) SUC C3. (**b**) SUC C4. (**c**) SUC C5. (**d**) SUC C6. (**e**) SUC C7. (**f**) SUC C8.

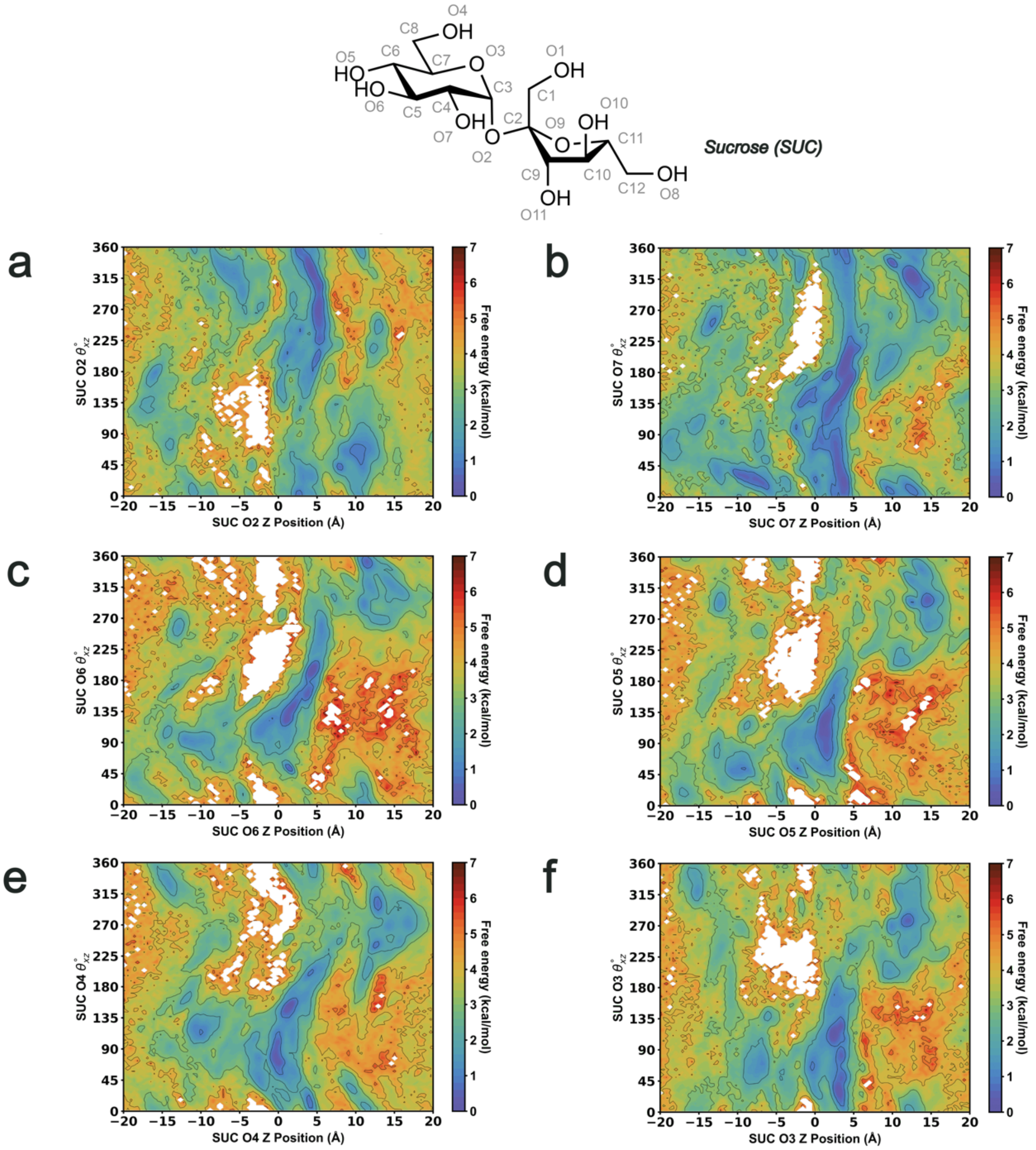

Supplementary Figure 21. MSM-weighted θ_{xz} analysis versus AtSWEET13 transmembrane channel Z position of the closest SUC molecule glucosyl oxygen atom to the Trp58-Trp180 binding pocket. (**a**) SUC O2. (**b**) SUC O7. (**c**) SUC O6. (**d**) SUC O5. (**e**) SUC O4. (**f**) SUC O3.

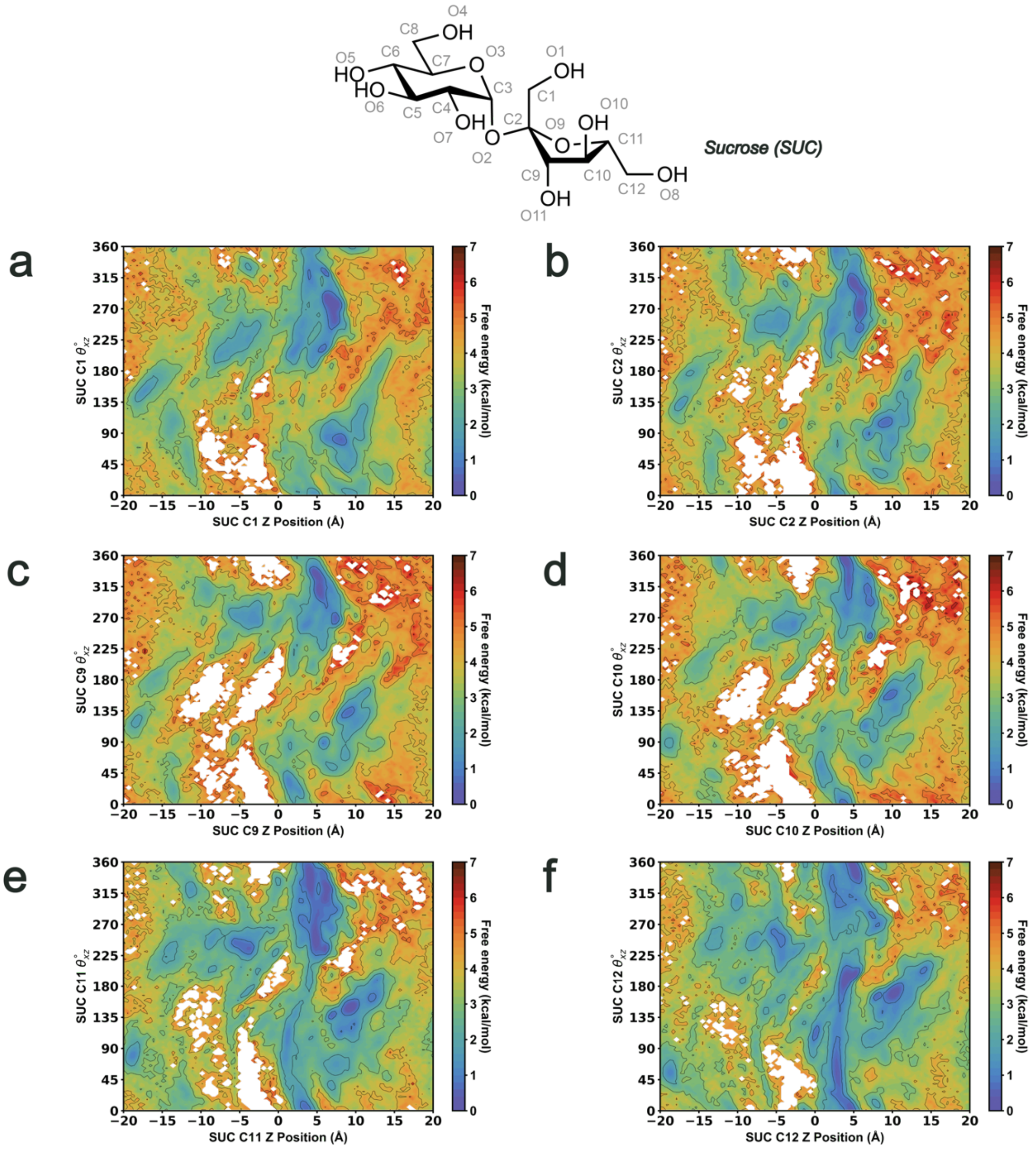

Supplementary Figure 22. MSM-weighted θ_{xz} analysis versus AtSWEET13 transmembrane channel Z position of the closest SUC molecule fructosyl carbon atom to the Trp58-Trp180 binding pocket. (**a**) SUC C1. (**b**) SUC C2. (**c**) SUC C9. (**d**) SUC C10. (**e**) SUC C11. (**f**) SUC C12.

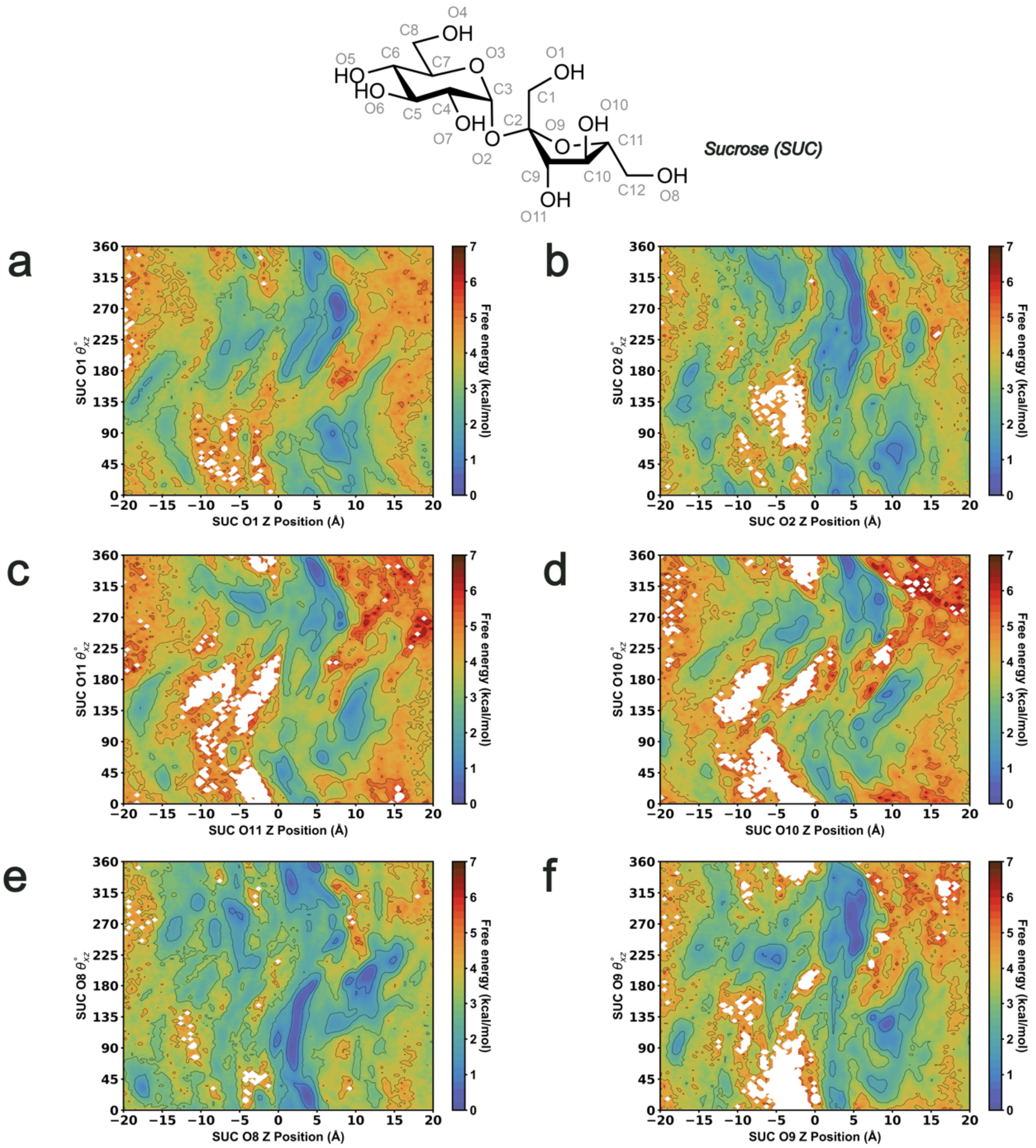

Supplementary Figure 23. MSM-weighted $\boldsymbol{\theta}_{xz}$ analysis versus AtSWEET13 transmembrane channel Z position of the closest SUC molecule fructosyl oxygen atom to the Trp58-Trp180 binding pocket. (**a**) SUC O1. (**b**) SUC O2. (**c**) SUC O11. (**d**) SUC O10. (**e**) SUC O8. (**f**) SUC O9.

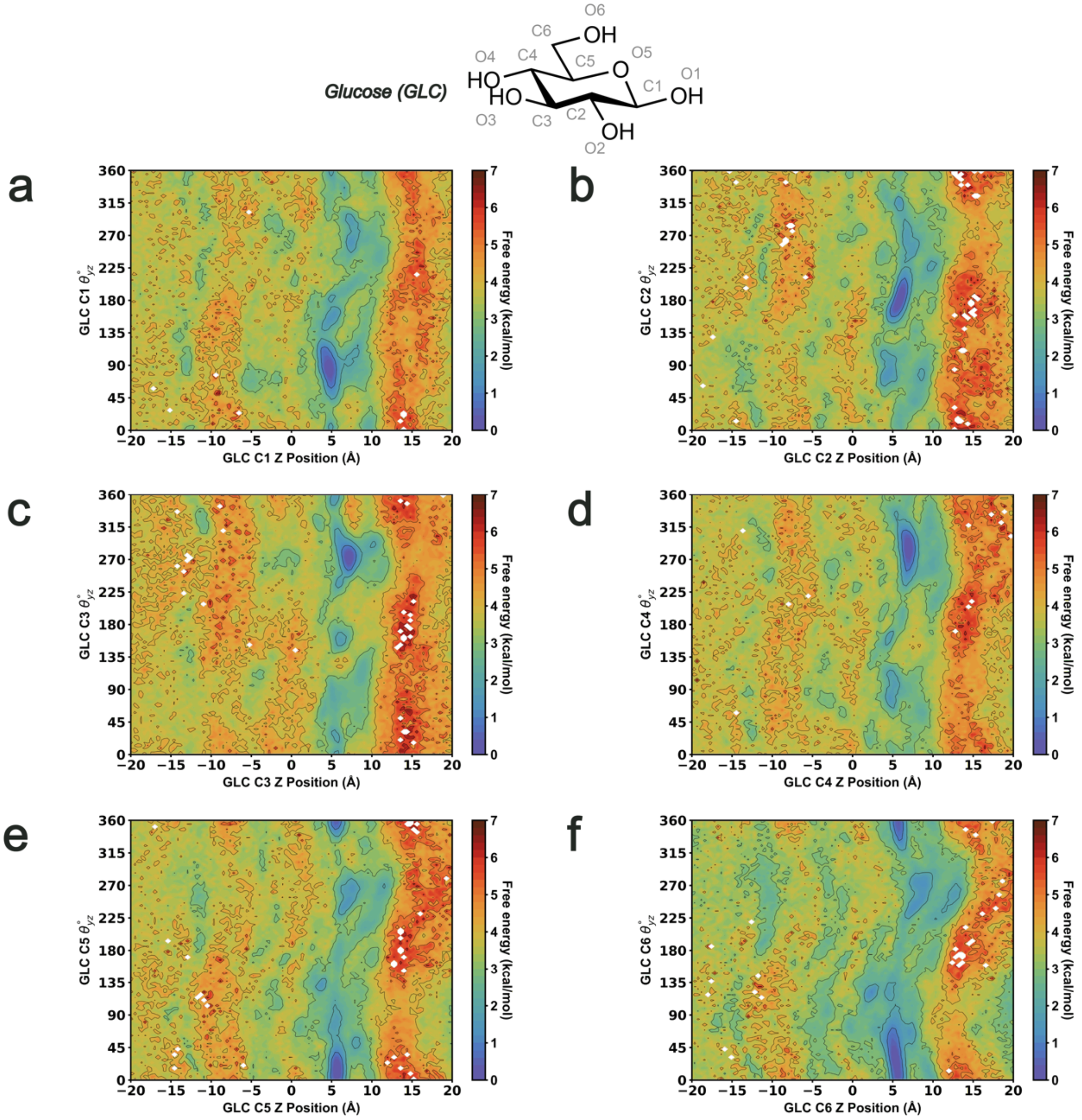

Supplementary Figure 24. MSM-weighted θ_{yz} analysis versus AtSWEET13 transmembrane channel Z position of the closest GLC molecule carbon atom to the Trp58-Trp180 binding pocket. (**a**) GLC C1. (**b**) GLC C2. (**c**) GLC C3. (**d**) GLC C4. (**e**) GLC C5. (**f**) GLC C6.

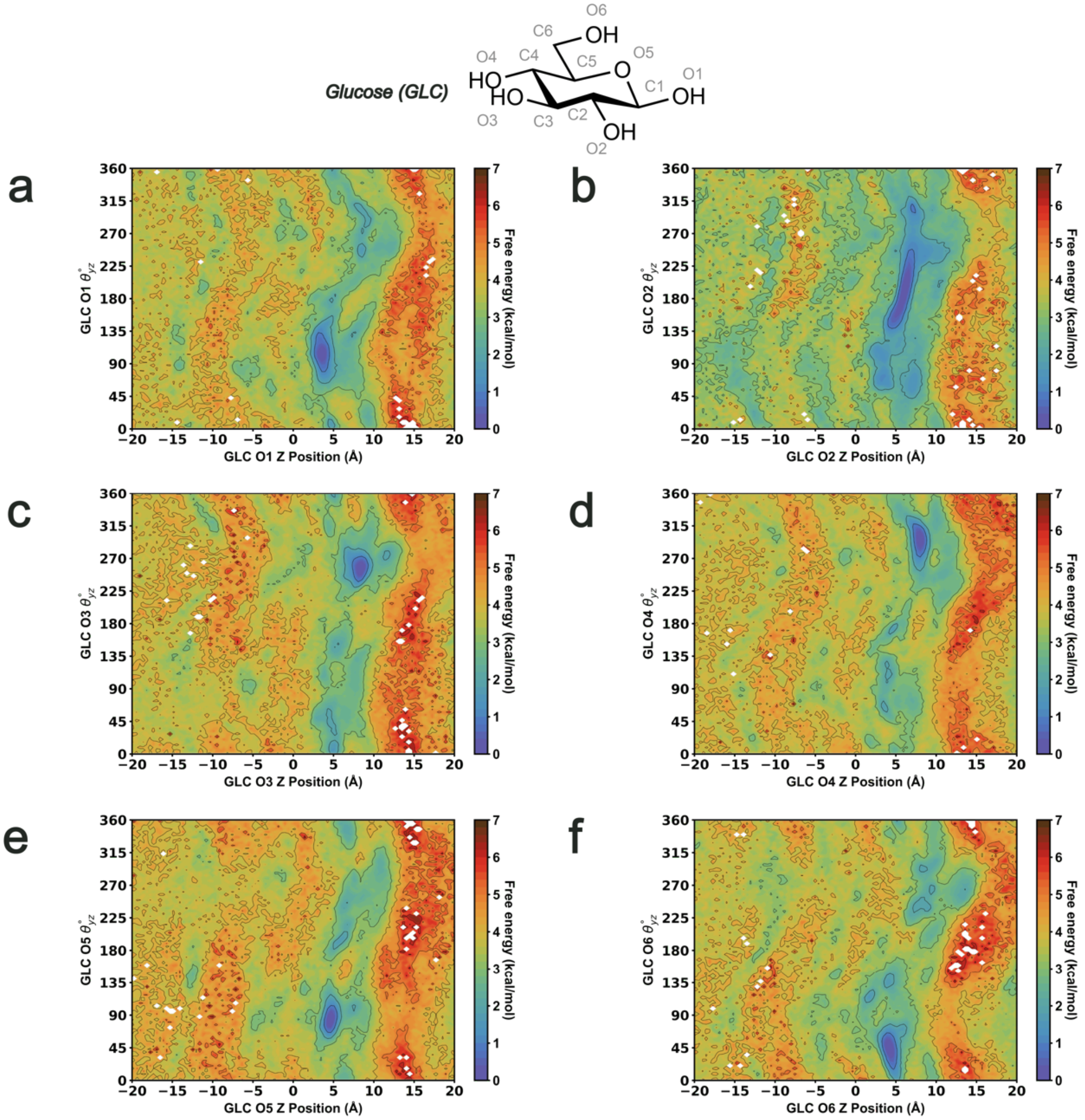

Supplementary Figure 25. MSM-weighted θ_{yz} analysis versus AtSWEET13 transmembrane channel Z position of the closest GLC molecule oxygen atom to the Trp58-Trp180 binding pocket. (**a**) GLC O1. (**b**) GLC O2. (**c**) GLC O3. (**d**) GLC O4. (**e**) GLC O5. (**f**) GLC O6.

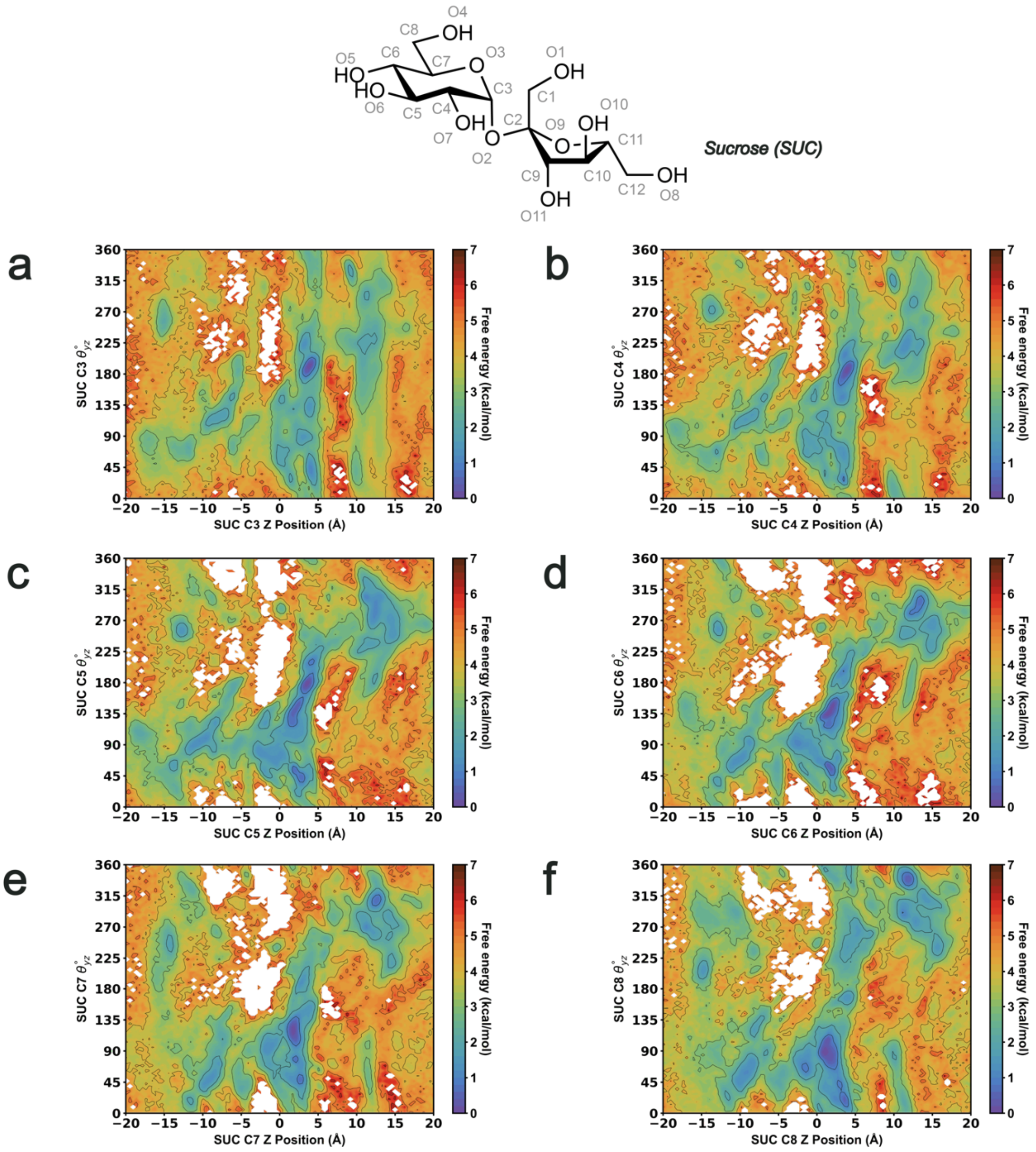

Supplementary Figure 26. MSM-weighted θ_{yz} analysis versus AtSWEET13 transmembrane channel Z position of the closest SUC molecule glucosyl carbon atom to the Trp58-Trp180 binding pocket. (**a**) SUC C3. (**b**) SUC C4. (**c**) SUC C5. (**d**) SUC C6. (**e**) SUC C7. (**f**) SUC C8.

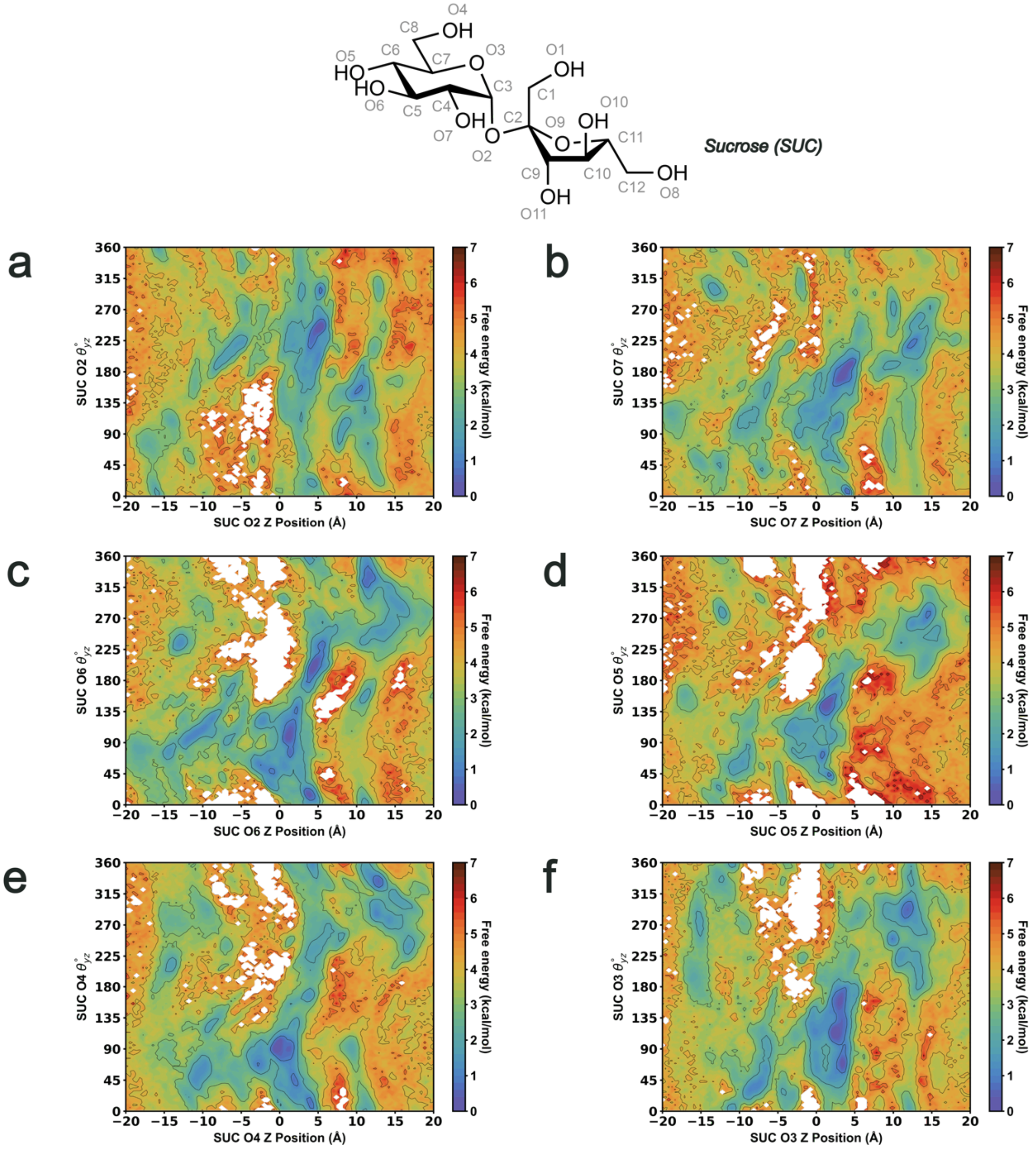

Supplementary Figure 27. MSM-weighted θ_{yz} analysis versus AtSWEET13 transmembrane channel Z position of the closest SUC molecule glucosyl oxygen atom to the Trp58-Trp180 binding pocket. (**a**) SUC O2. (**b**) SUC O7. (**c**) SUC O6. (**d**) SUC O5. (**e**) SUC O4. (**f**) SUC O3.

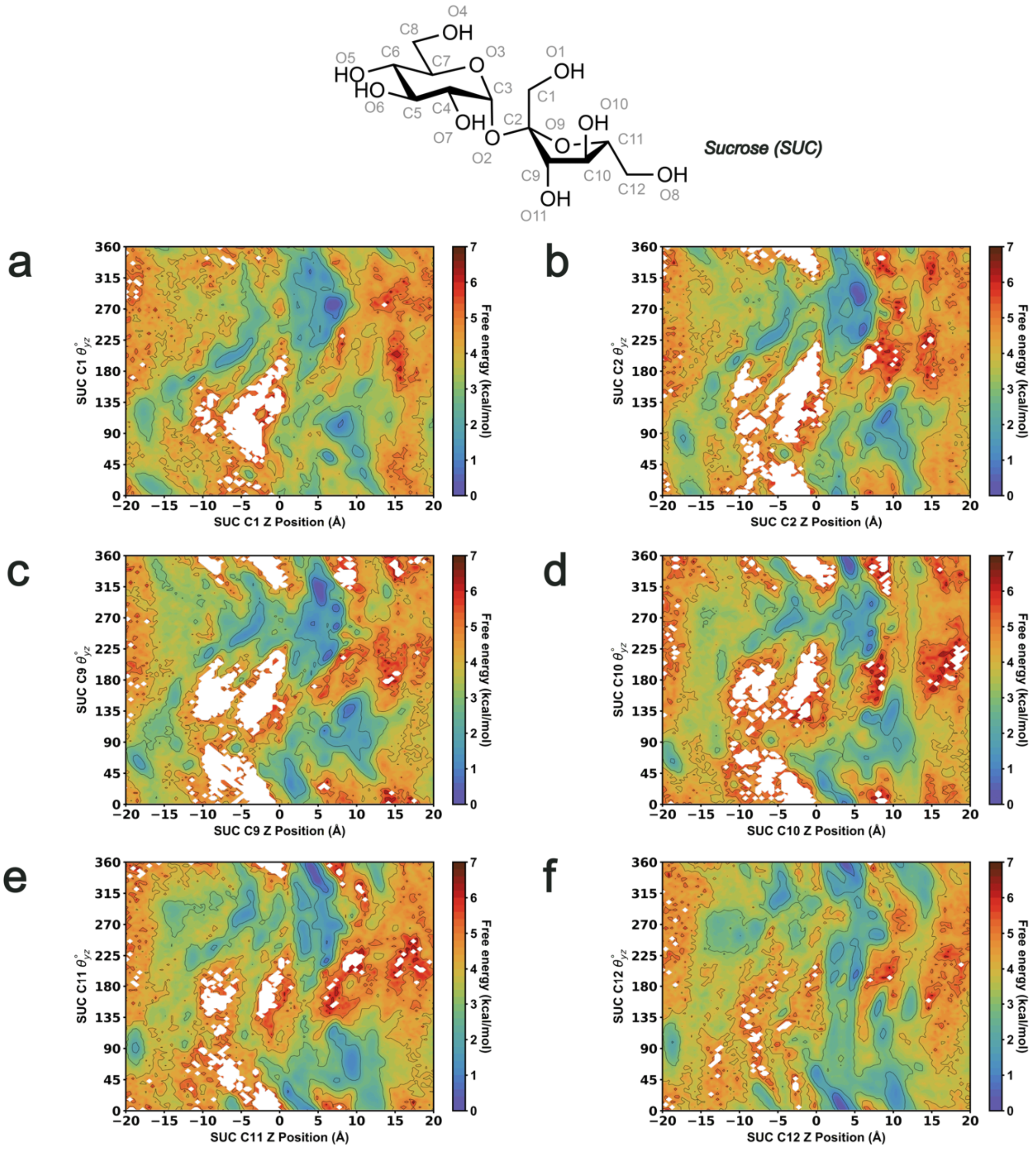

Supplementary Figure 28. MSM-weighted θ_{yz} analysis versus AtSWEET13 transmembrane channel Z position of the closest SUC molecule fructosyl carbon atom to the Trp58-Trp180 binding pocket. (**a**) SUC C1. (**b**) SUC C2. (**c**) SUC C9. (**d**) SUC C10. (**e**) SUC C11. (**f**) SUC C12.

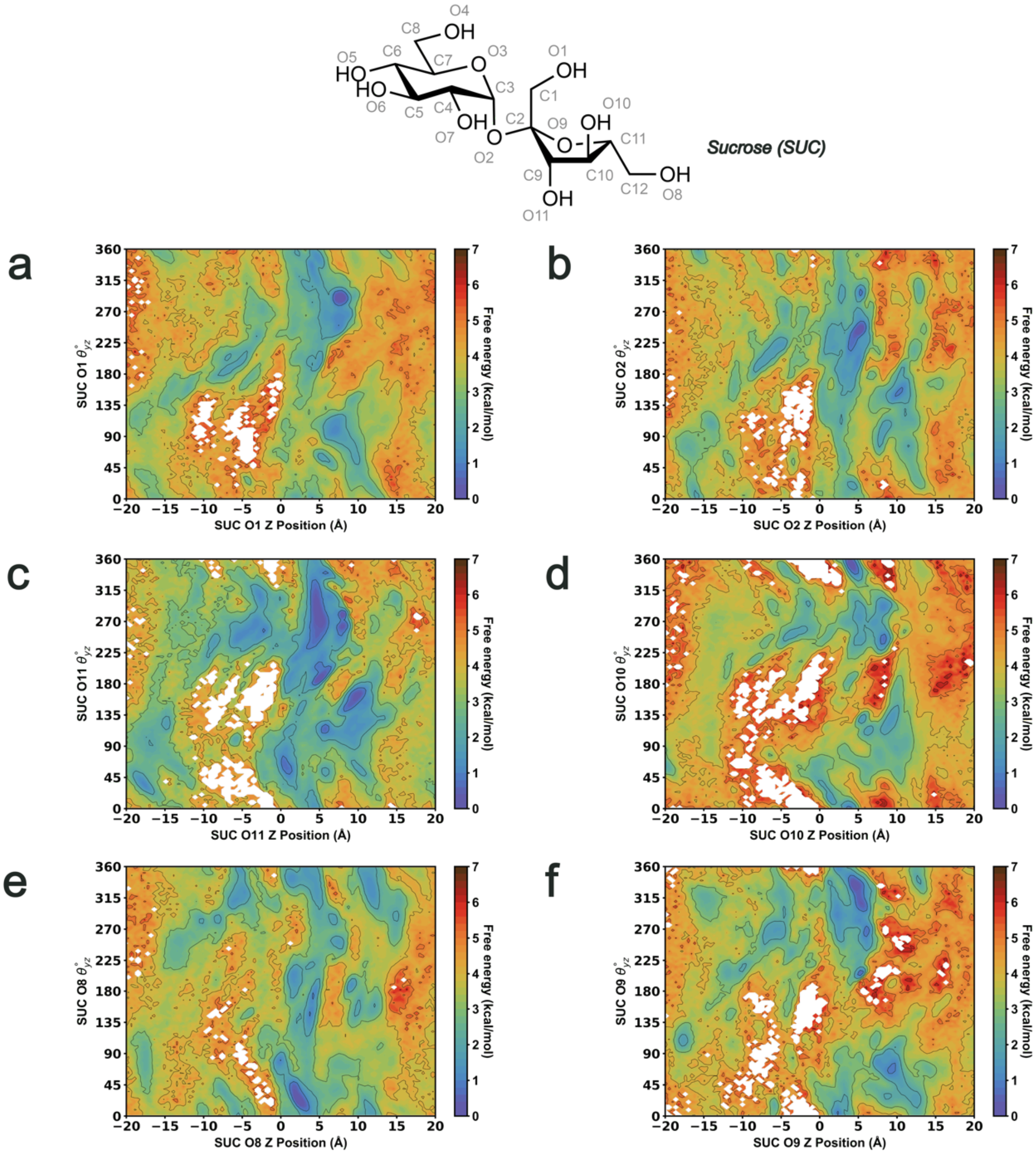

Supplementary Figure 29. MSM-weighted θ_{yz} analysis versus AtSWEET13 transmembrane channel Z position of the closest SUC molecule fructosyl oxygen atom to the Trp58-Trp180 binding pocket. (**a**) SUC O1. (**b**) SUC O2. (**c**) SUC O11. (**d**) SUC O10. (**e**) SUC O8. (**f**) SUC O9.

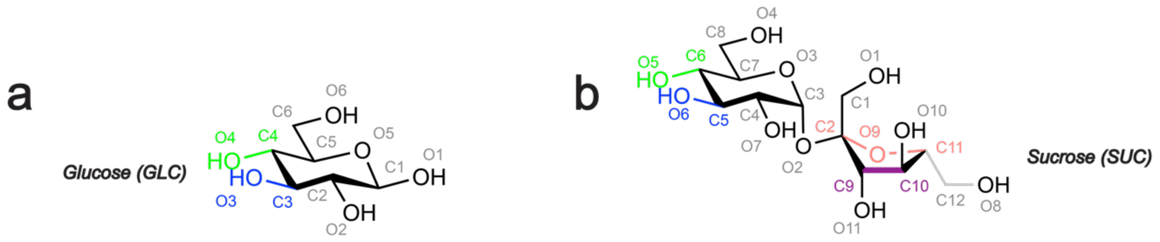

Supplementary Figure 30. (**a**) GLC and (**b**) SUC structures with highlighted functional considered critical for molecular recognition by AtSWEET13. Atoms colored blue and green represent moieties which AtSWEET13 recognizes as "GLC-like" between GLC and SUC. Atoms colored salmon, grey, and magenta represent moieties which AtSWEET13 recognizes as "SUC-like". The advent of the fructosyl monomeric unit of SUC is best represented by the salmon, grey, and magenta moieties on SUC. Classification of these functional units was based off rational interpretation of atom-specific θ rotation analyses.

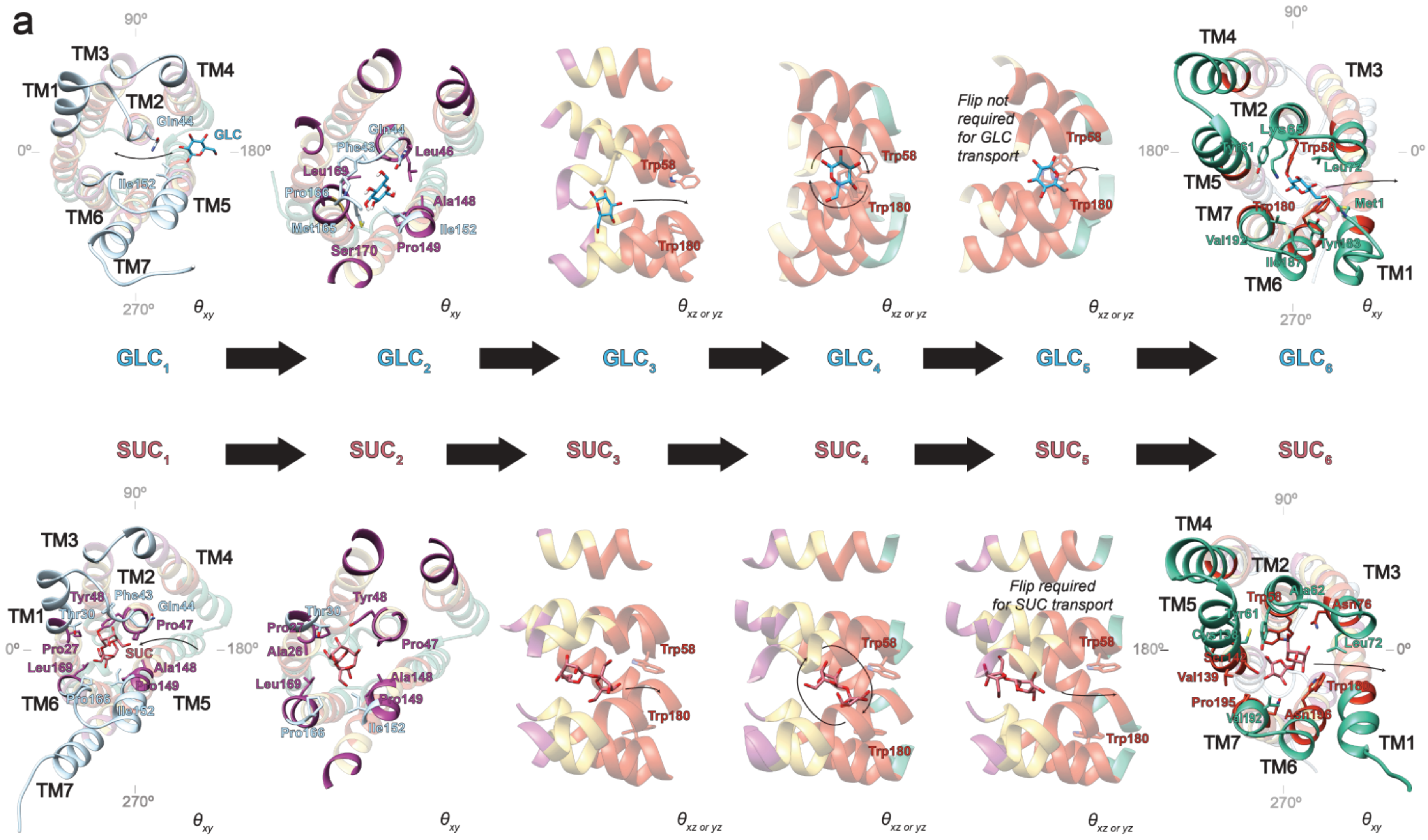

Supplementary Figure 31. Discriminative sugar transport events for AtSWEET13. States are shown for (**a**) GLC transport and (**b**) SUC transport. Each of the numbered states correspond to those enumerated in Main Text Figure 4b. Each snapshot is accompanied by a marker for indicating which type of rotational analysis the structure is intended to convey.

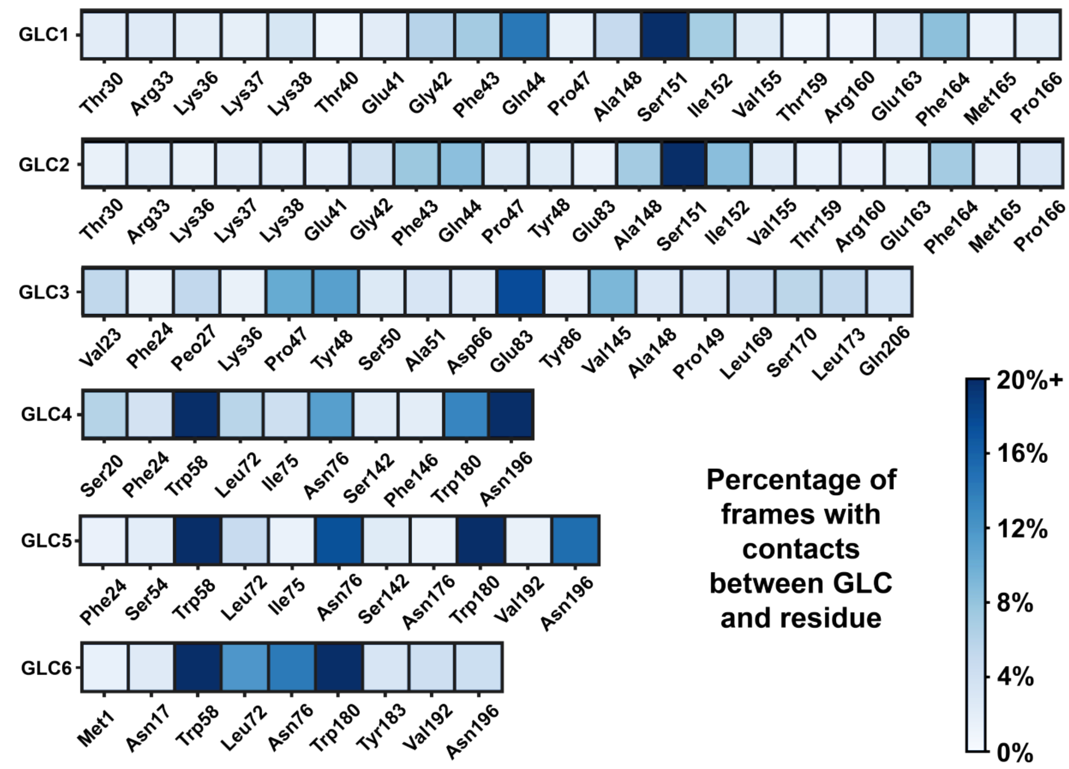

Supplementary Figure 32. GetContacts analysis for representative frames of metastable states throughout GLC transport. Metastable states are the same as those enumerated from Main Text Figure 4b.

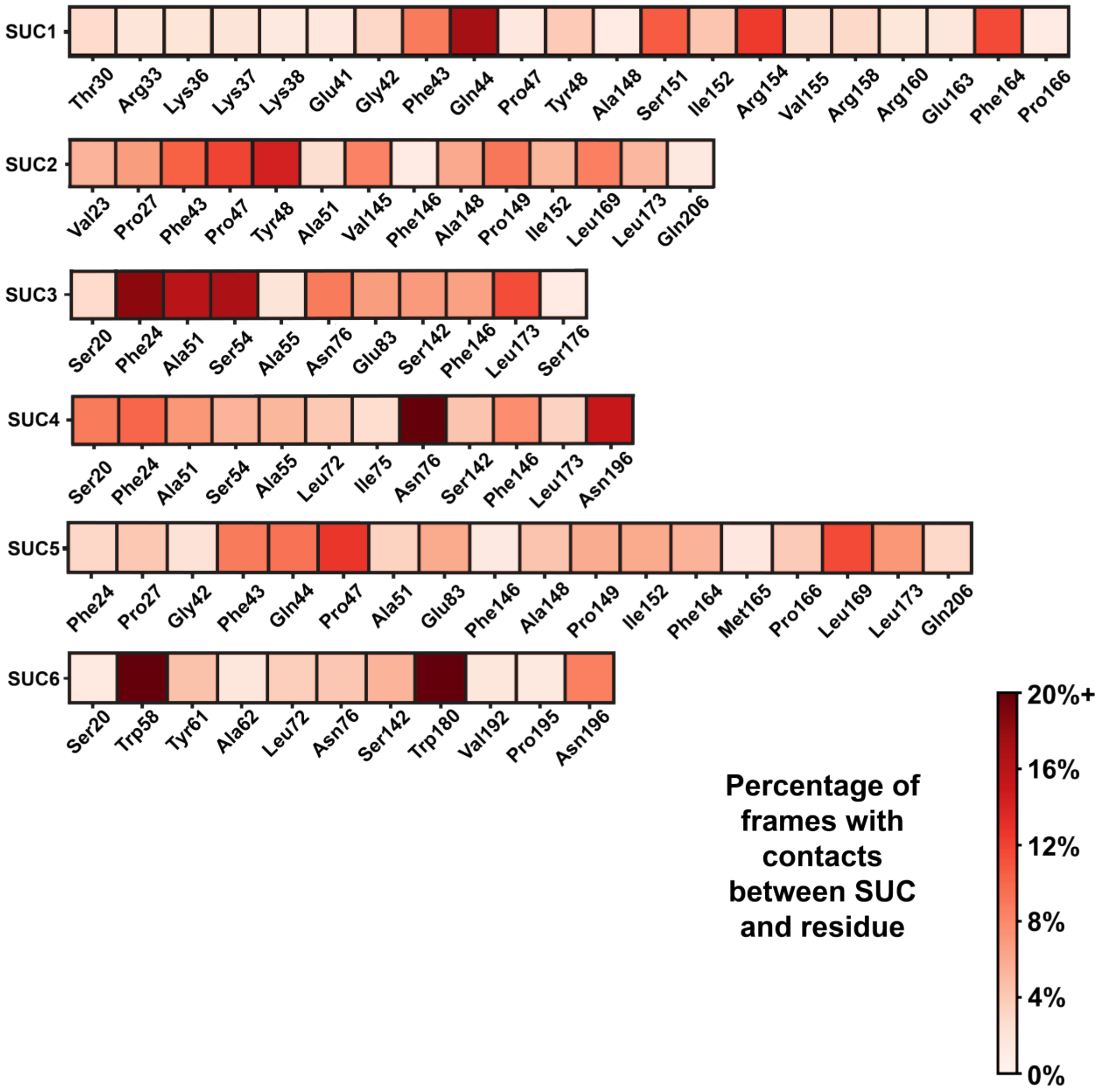

Supplementary Figure 33. GetContacts analysis for representative frames of metastable states throughout SUC transport. Metastable states are the same as those enumerated from Main Text Figure 4b.

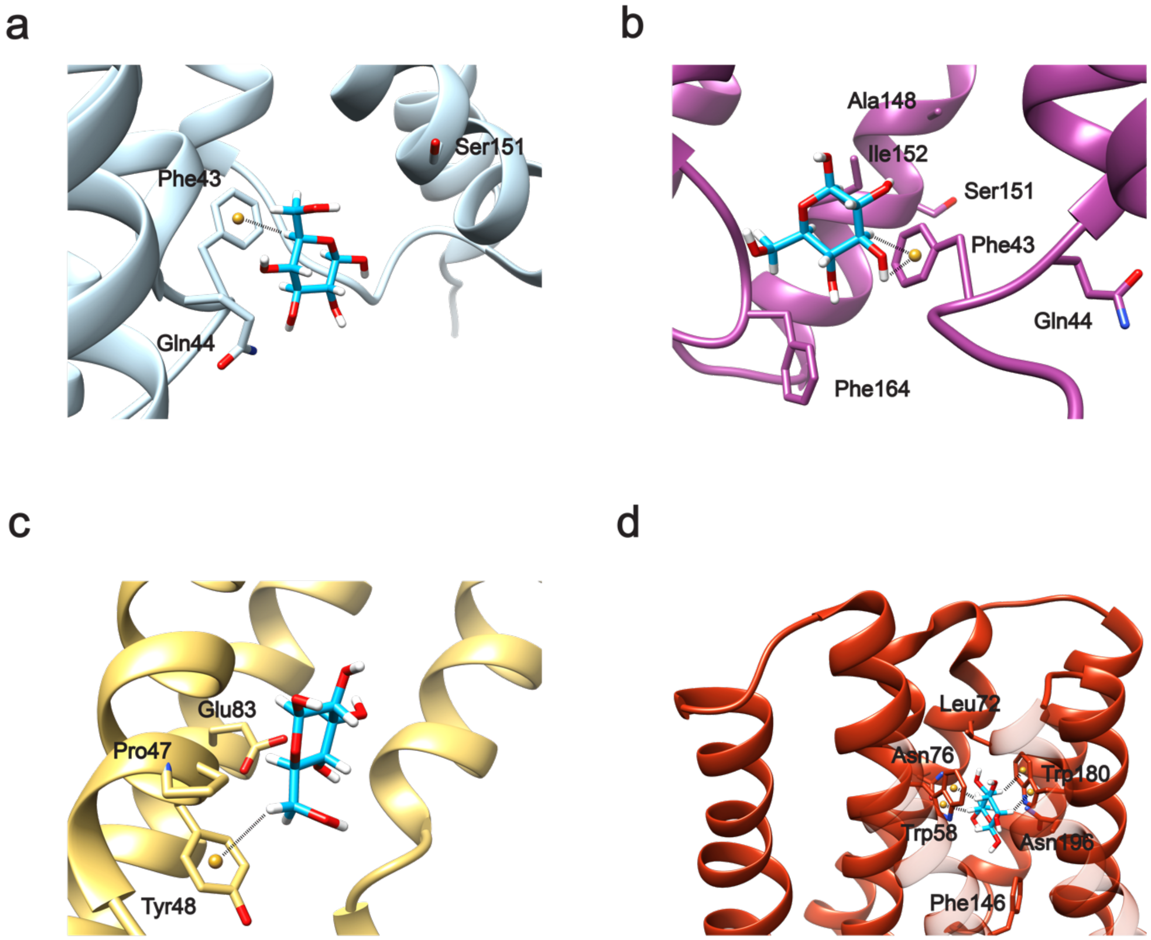

Supplementary Figure 34. C-H···π stacking and other interactions between GLC and AtSWEET13 throughout molecular recognition and transport. (**a**) GLC binding during early IF recognition along intracellular gate residue Phe43. (**b**) GLC-Phe43 interactions facilitate further substrate entry into the transmembrane channel. (**c**) Preparative GLC binding interactions during an extended HG-IF-like state before commitment to alternate access. (**d**) Extensive GLC C-H···π stacking with the Trp58-Trp180 binding pocket. Goldenrod spheres are used to represent the centers of mass of aromatic systems within residue side chains. C-H···π stacking is indicated using vertical black bars between substrate hydrogen atoms and the aromatic goldenrod spheres.

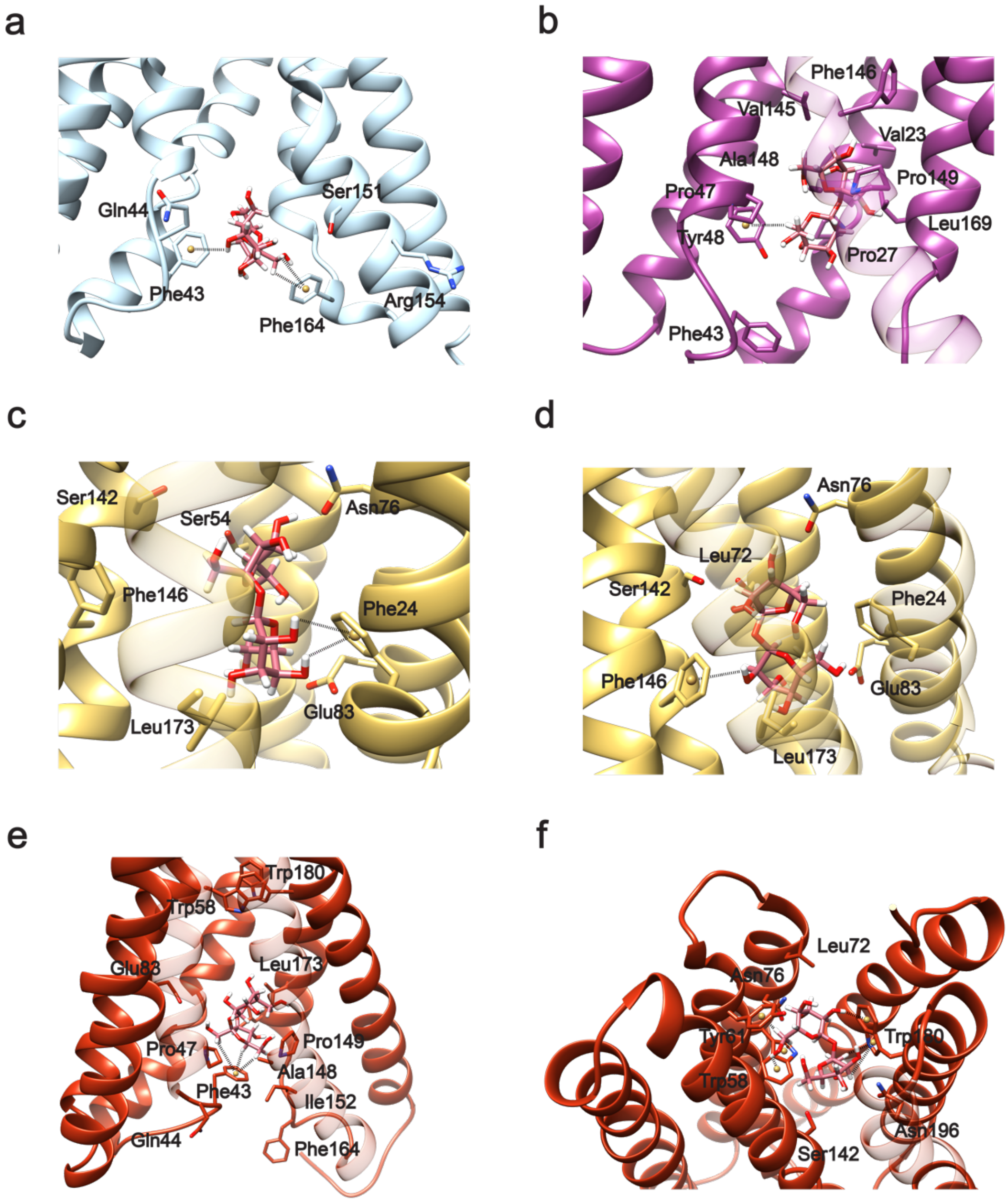

Supplementary Figure 35. C-H···π stacking and other interactions between SUC and AtSWEET13 residues throughout molecular recognition and transport. (**a**) SUC binding during early IF recognition along intracellular gate residues Phe43 and Phe164. (**b**) Further SUC entry into the intracellular-facing vestibule of the transmembrane channel. Preparative SUC binding interactions during an extended HG-IF-like are stabilized by C-H···π stacking with either (**c**) Phe24 or (**d**) Phe146. (**e**) SUC-Phe43 C-H···π stacking maintains an HG conformation during required facial inversion. (**f**) SUC enjoys extensive C-H···π stacking throughout commitment to alternate access. Goldenrod spheres are used to represent the centers of mass of aromatic systems within residue side chains. C-H···π stacking is indicated using vertical black bars between substrate hydrogen atoms and the aromatic goldenrod spheres.

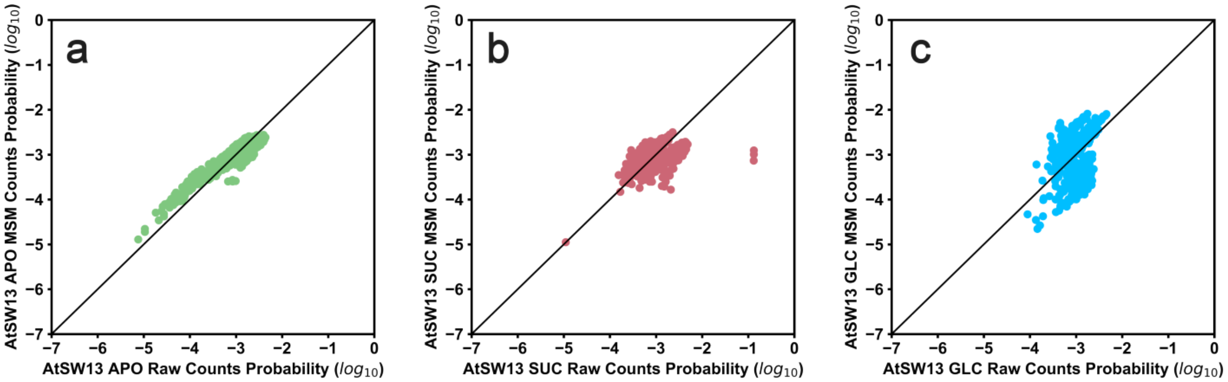

Supplementary Figure 36. Raw counts versus MSM population in each clustered state for (**a**) *Apo*, (**b**) SUC, and (**c**) GLC transport by AtSWEET13.

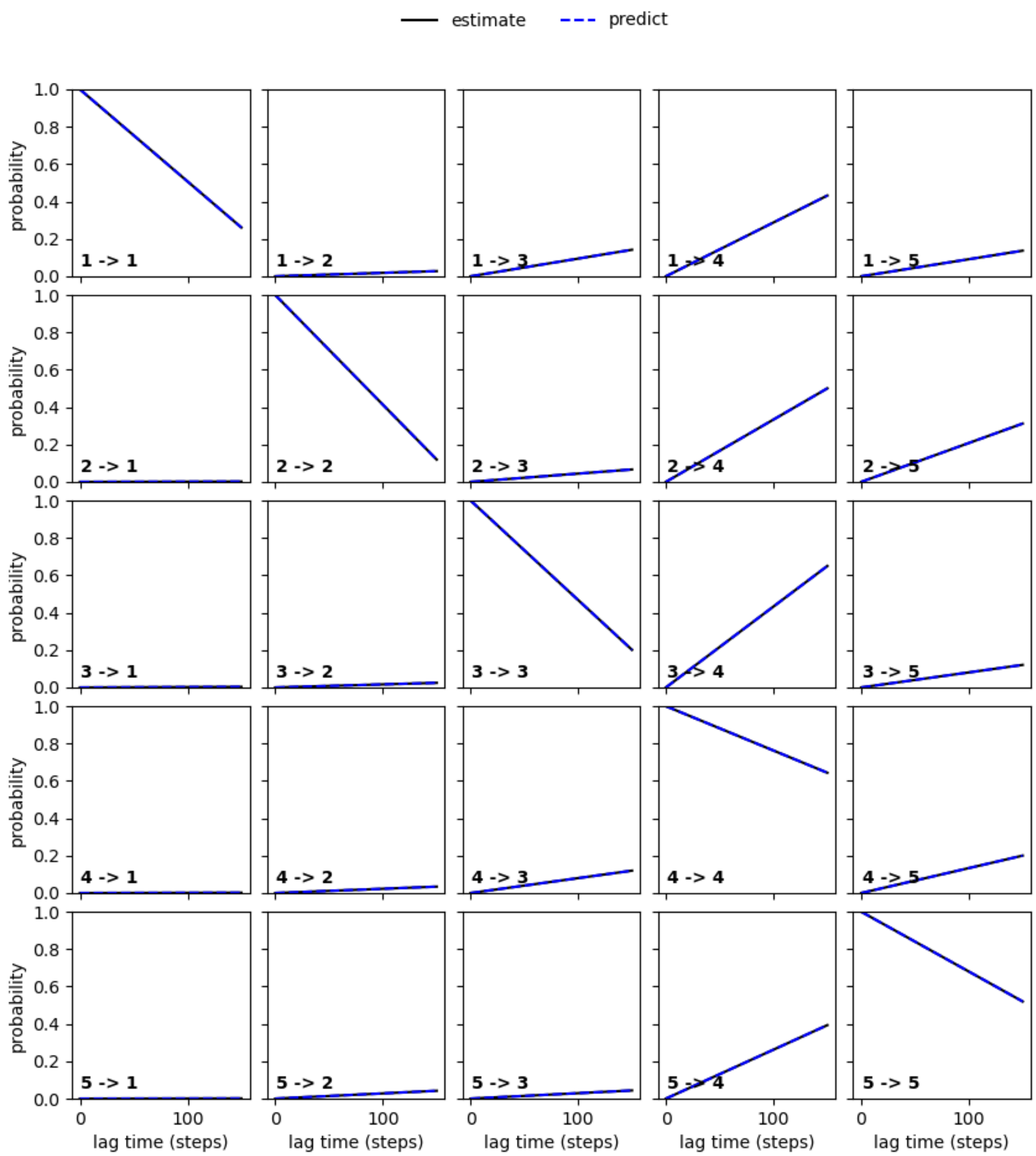

Supplementary Figure 37. Chapman-Kolmogorov test for the MSM of *apo* AtSWEET13 conformational dynamics and transport. The MSM lagtime is 30 ns (150 steps).

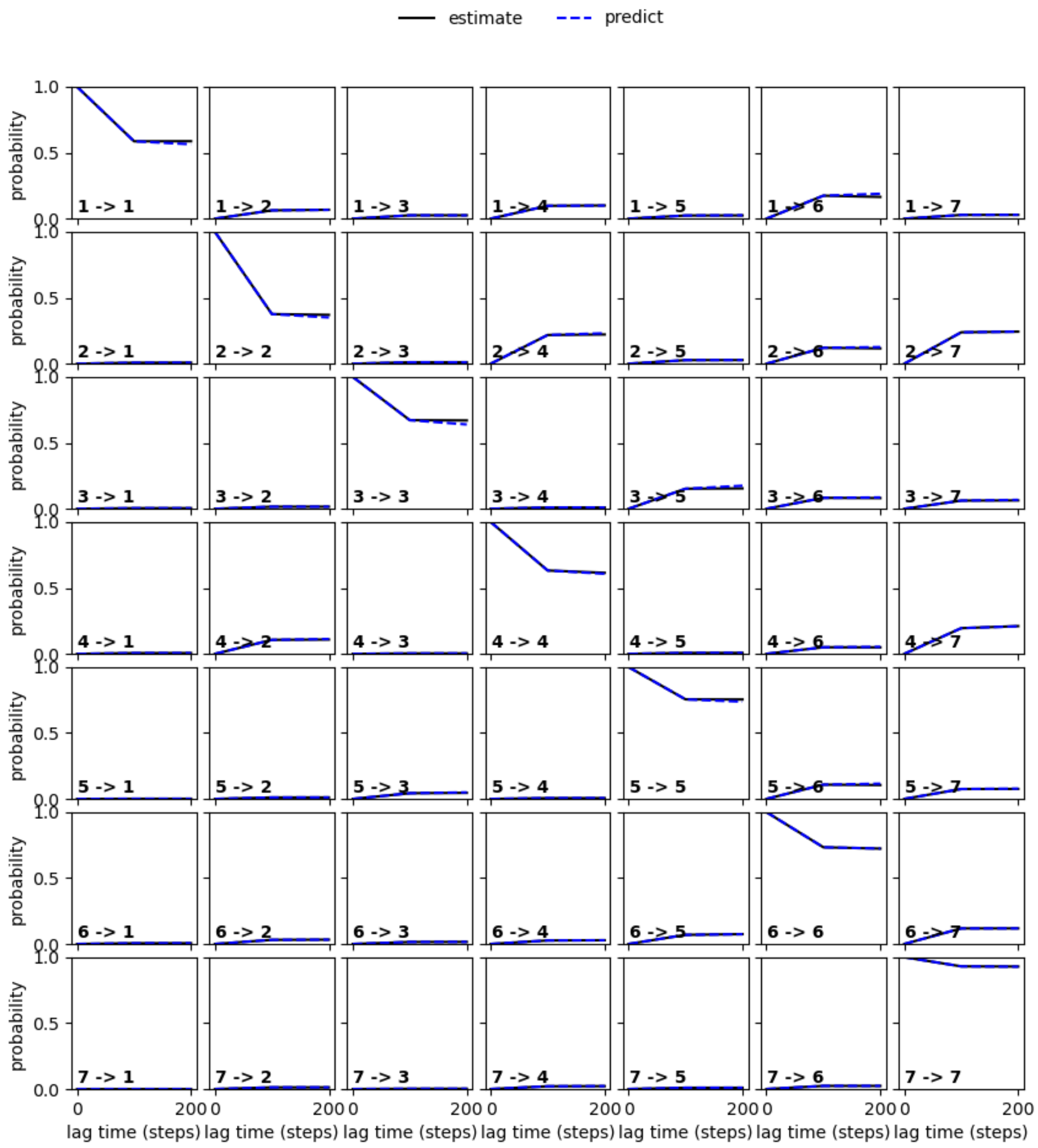

Supplementary Figure 38. Chapman-Kolmogorov test for the MSM of AtSWEET13 conformational dynamics during GLC transport. The MSM lagtime is 20 ns (100 steps).

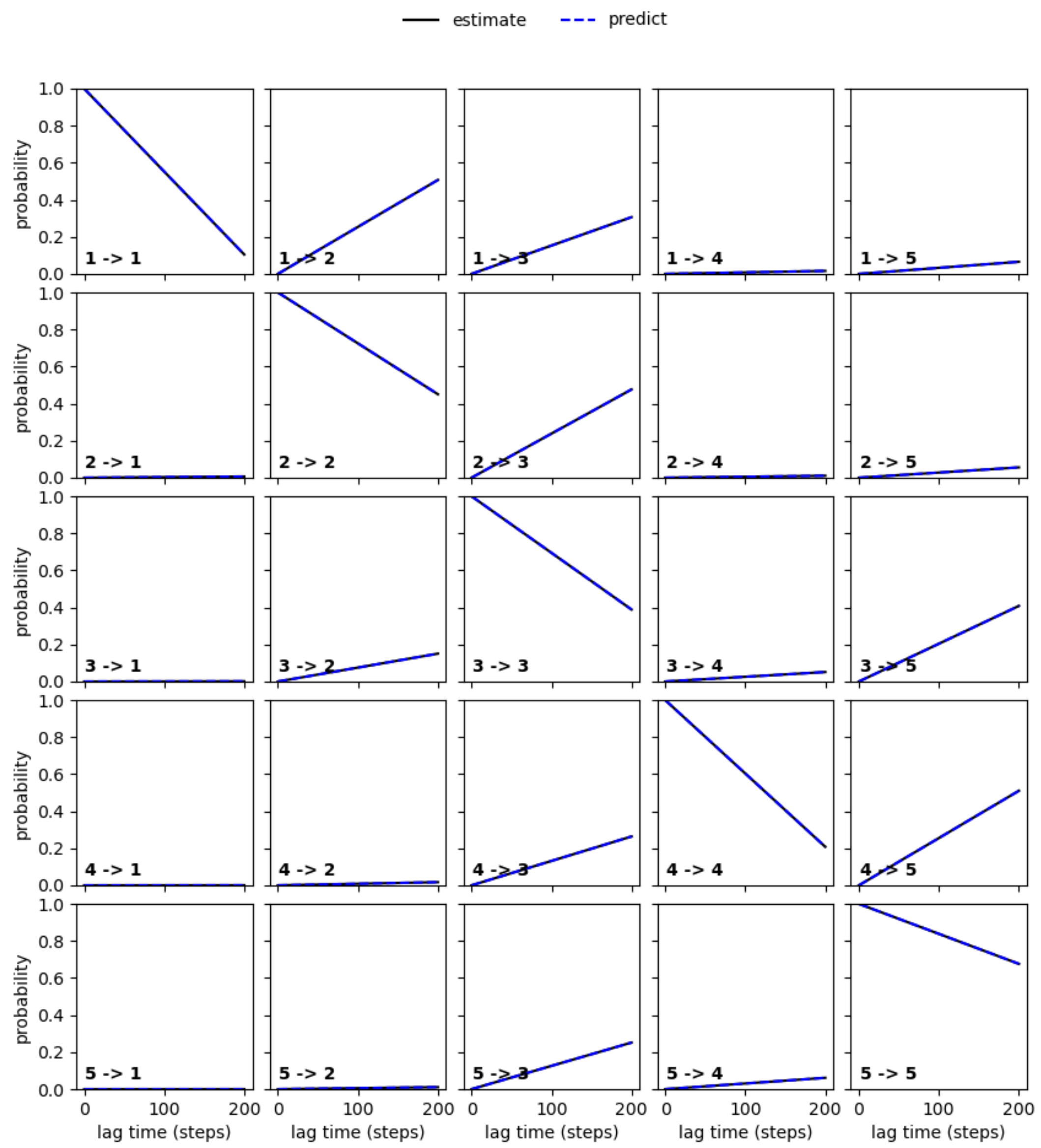

Supplementary Figure 39. Chapman-Kolmogorov test for the MSM of AtSWEET13 conformational dynamics during SUC transport. The MSM lagtime is 40 ns (200 steps).

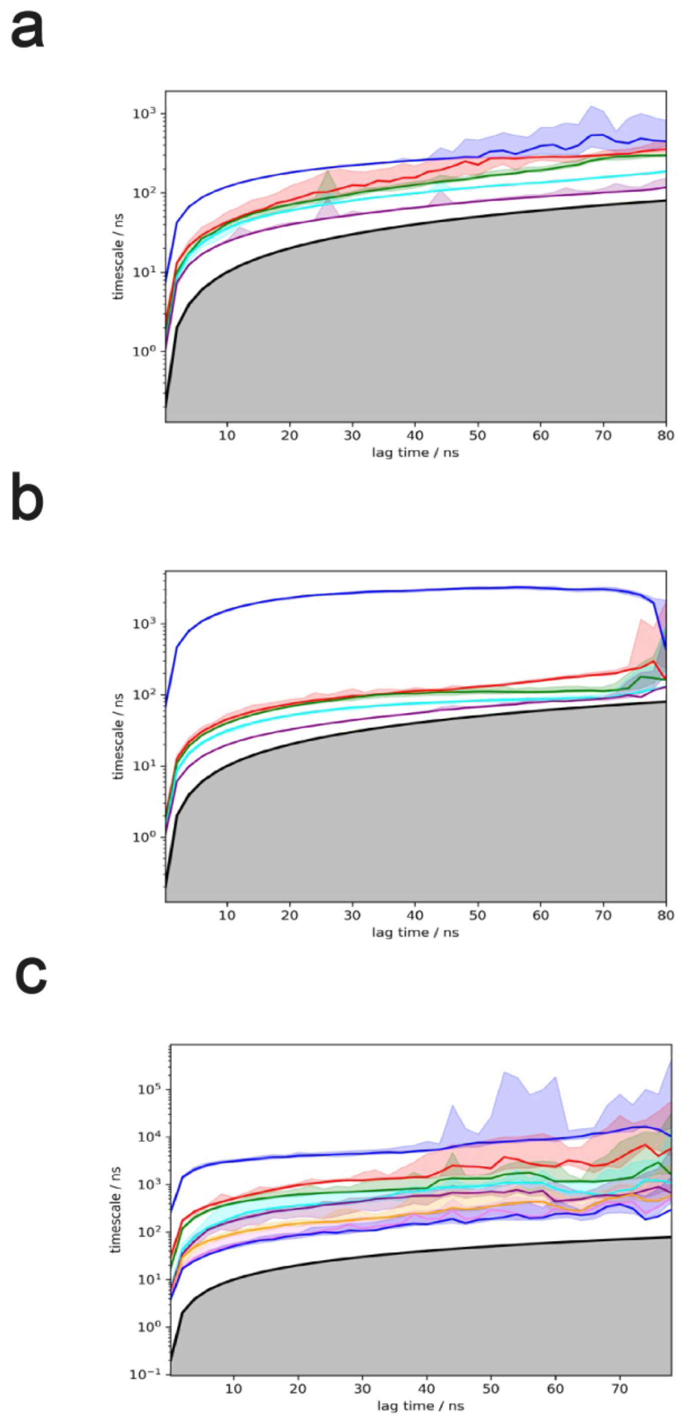

Supplementary Figure 40. Implied timescale plots calculated with Bayesian error for (**a**) *Apo*, (**b**) SUC and (**c**) GLC transport.

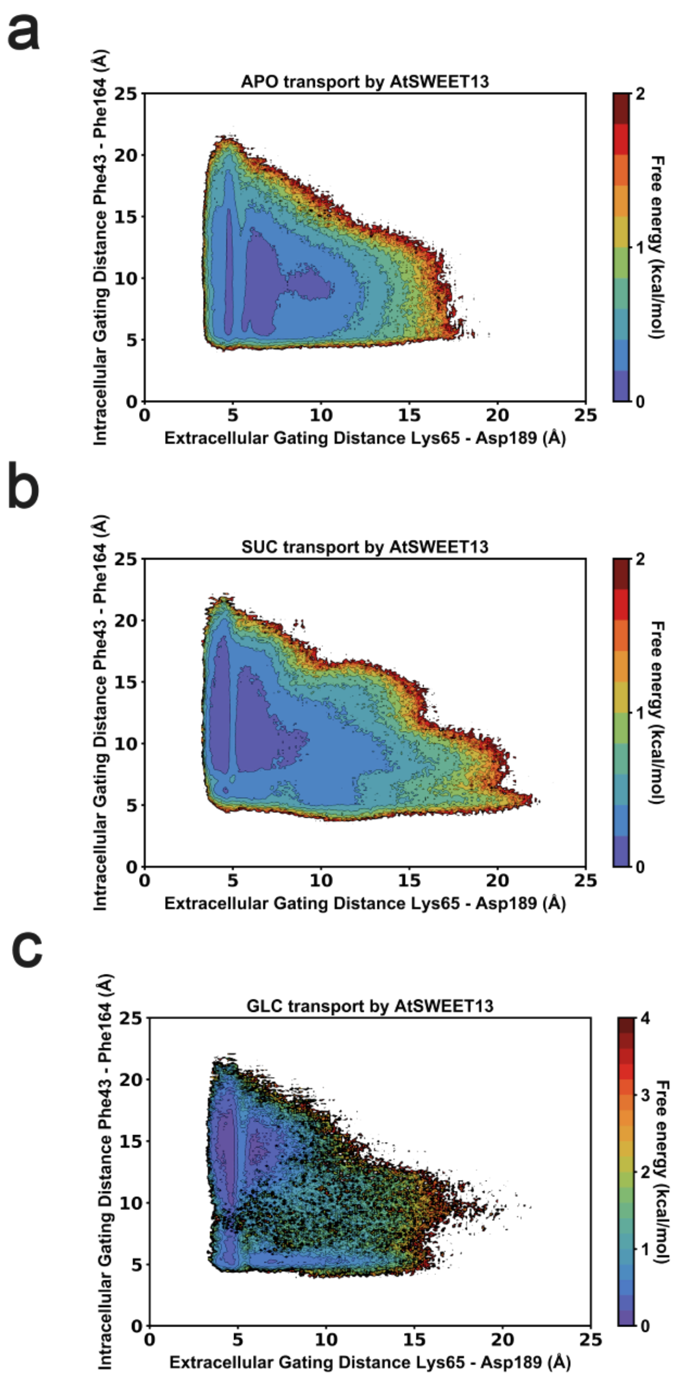

Supplementary Figure 41. MSM-weighted bootstrap error plots for adaptive sampling of gating landscapes. (**a**) *Apo*, (**b**) SUC, and (**c**) GLC. Colorbar scales vary between plots to improve landscape resolution and aid in visualization.

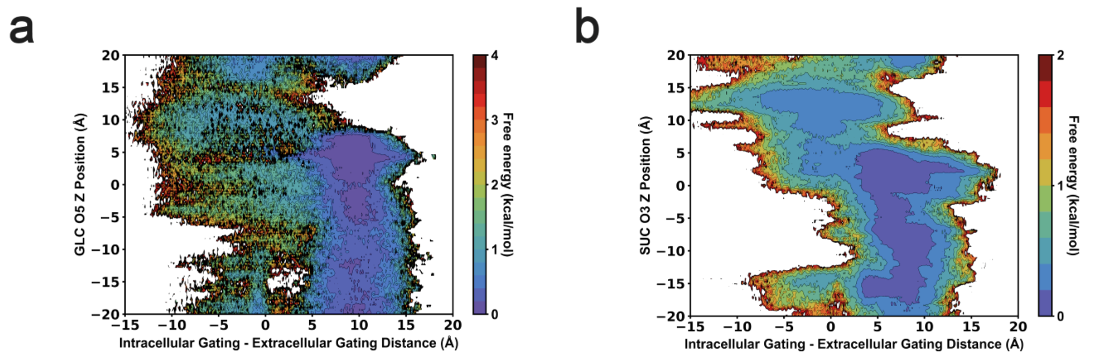

Supplementary Figure 42. MSM-weighted bootstrap error plots for adaptive sampling of (**a**) SUC and (**b**) GLC intracellular minus extracellular gating distance versus AtSWEET13 transmembrane channel ligand Z position landscapes. Colorbar scales vary between plots to improve landscape resolution and aid in visualization.

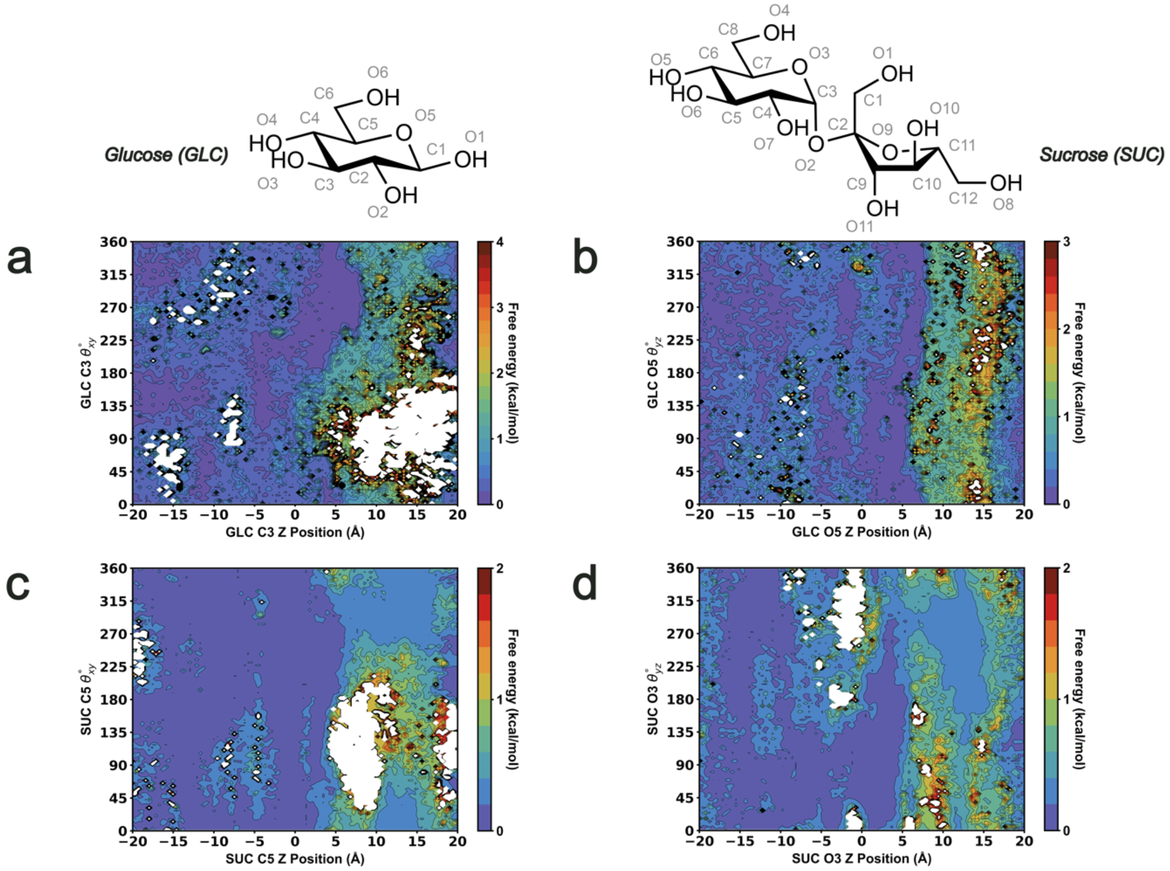

Supplementary Figure 43. MSM-weighted bootstrap error plots for adaptive sampling of $\boldsymbol{\theta}$ rotation analyses presented in Main Text Figure 4. (**a**) GLC C3 $\boldsymbol{\theta}_{xy}$. (**b**) GLC O5 $\boldsymbol{\theta}_{yz}$. (**c**) SUC C5 $\boldsymbol{\theta}_{xy}$. (**d**) SUC O3 $\boldsymbol{\theta}_{yz}$. Colorbar scales vary between plots to improve landscape resolution and aid in visualization.

Supplementary Table 1. Finalized features used for MSM discretization. Residue-residue distances are signified as “RES#1_ATOM_RES#2_ATOM”. Ligand center of mass (CoM) and atom are listed for either Z position or a specific theta (***θ***) vector.

	**APO**	**SUC**	**GLC**
**Final MSM features for trajectory discretization**	GLY42_CA_GLU163_CB	GLY16_CA_LEU71_CB	GLY13_CA_LEU71
	PRO47_CB_LEU169_CB	GLY13_CA_LEU71_CB	GLY16_CA_LEU71_CB
	GLY42_CA_PHE164_CB	ILE75_CB_GLY16_CA	ILE75_CB_GLY16_CA
	PHE43_CB_PHE164_CB	LYS65_CB_ASP189_CB	GLY42_CA_THR30_CB
	PRO166_CB_PRO47_CB	LYS65_CB_ILE187_CB	GLY42_CA_PRO166_CB
		THR68_CB_ILE187_CB	THR40_CB_PHE43_CB
		SUC CoM Z position	THR68_CB_ILE187_CB
		SUC_C2_theta_xy	PHE43_CB_LEU169_CB
		SUC_C2_theta_xz	GLU41_CB_ARG33_CB
		SUC_C2_theta_yz	ARG33_CB_THR30_CB
		SUC_C5_theta_xy	GLU41_CB_THR30_CB
		SUC_C5_theta_xz	GLU41_CB_ILE34_CB
		SUC_C5_theta_yz	GLY42_CA_MET165_CB
		SUC_C6_theta_xy	GLC CoM Z position
		SUC_C6_theta_xz	
		SUC_C6_theta_yz	
		SUC_C9_theta_xy	
		SUC_C9_theta_xz	
		SUC_C9_theta_yz	
		SUC_C10_theta_xy	
		SUC_C10_theta_xz	
		SUC_C10_theta_yz	
		SUC_C11_theta_xy	
		SUC_C11_theta_xz	
		SUC_C11_theta_yz	
		SUC_C12_theta_xy	
		SUC_C12_theta_xz	
		SUC_C12_theta_yz	
		SUC_O5_theta_xy	
		SUC_O5_theta_xz	
		SUC_O5_theta_yz	
		SUC_O6_theta_xy	
		SUC_O6_theta_xz	
		SUC_O6_theta_yz	
		SUC_O9_theta_xy	
		SUC_O9_theta_xz	
		SUC_O9_theta_yz	

Supplementary Table 2. Descriptor correlation to tIC1 from feature-diverse tICA decomposition of GLC transport.

System	GLC
**Feature**	**tIC1 correlation | *r* |**
GLY13-LEU71	0.9030
GLY16-LEU71	0.8950
ILE75-GLY16	0.8923
GLY42-THR30	0.8654
GLY42-PRO166	0.8318
THR40-PHE43	0.8229
THR68-ILE187	0.7994
PHE43-LEU169	0.7629
GLU41-ARG33	0.7502
ARG33-THR30	0.7466
GLU41-THR30	0.7394
GLU41-ILE34	0.7258
GLY42-MET165	0.7175

Supplementary Table 3. Descriptor correlation to tIC1 from feature-diverse tICA decomposition of SUC transport.

System	SUC	System	SUC	System	SUC	System	SUC
Feature	tIC1 correlation | *r* |	Feature	tIC1 correlation | *r* |	Feature	tIC1 correlation | *r* |	Feature	tIC1 correlation | *r* |
GLY16-LEU71	0.9148	ILE152-O5	0.7895	ASN5-O5	0.7507	ARG160-O5	0.7178
GLY13-LEU71	0.9026	TYR210-C5	0.7890	GLY135-C5	0.7480	LYS132-C12	0.7172
ILE75-GLY16	0.8937	LEU209-O6	0.7866	LEU72-C5	0.7475	ASP189-C12	0.7151
ILE187-O5	0.8529	PHE213-C6	0.7850	LEU169-C6	0.7465	PRO47-O5	0.7147
GLU131-C6	0.8510	GLY13-O5	0.7845	PHE213-O6	0.7442	PHE90-C5	0.7142
ILE187-C6	0.8493	LYS65-ILE187	0.7837	TYR61-O6	0.7440	TYR86-O5	0.7137
LYS65-C6	0.8442	ALA62-C6	0.7825	ILE152-C5	0.7428	ALA2-C6	0.7135
LYS65-C5	0.8437	GLY13-O5	0.7819	GLY184-O5	0.7428	PRO47-C5	0.7114
GLY13-C5	0.8390	VAL162-O5	0.7809	LEU3-O6	0.7413	TYR48-O5	0.7094
GLU131-O5	0.8322	ASP189-C5	0.7760	PHE90-C6	0.7393	ASN5-C5	0.7091
ASP189-C6	0.8228	PRO149-C5	0.7753	GLY135-C12	0.7378	ARG160-C5	0.7088
LYS65-O6	0.8225	ALA62-O5	0.7722	PRO47-C6	0.7376	CYS138-C6	0.7085
ILE187-C5	0.8174	VAL192-C6	0.7712	TYR86-C6	0.7361	SER161-C6	0.7084
LYS65-O5	0.8166	ILE152-C6	0.7706	LEU169-O5	0.7335	PRO166-O5	0.7084
TYR61-C6	0.8162	THR68-ILE187	0.7701	LEU72-O6	0.7331	TYR86-C5	0.7060
GLU131-C5	0.8161	LEU3-C5	0.7692	PRO149-O6	0.7330	GLY135-C11	0.7056
GLY13-C6	0.8153	PHE213-C5	0.7681	VAL162-C5	0.7328	TYR183-C5	0.7052
LEU209-C6	0.8149	LEU72-O5	0.7667	GLU131-C12	0.7316	GLU163-O5	0.7051
ASP189-O5	0.8141	TYR183-O5	0.7667	ASN17-O6	0.7309	CYS138-O9	0.7045
LEU209-C5	0.8079	LYS132-C6	0.7663	ASN17-C5	0.7308	LYS65-O9	0.7031
LEU209-O5	0.8075	LEU3-C6	0.7655	THR68-C5	0.7306	LEU169-C5	0.7026
GLY135-C6	0.8053	PRO149-O5	0.7652	TYR48-C6	0.7297	ILE34-C6	0.7026
TYR210-C6	0.8039	LEU3-O5	0.7651	CYS138-C12	0.7292	VAL162-O6	0.7008
ALA62-C5	0.8009	TYR210-O6	0.7647	ALA2-O5	0.7272	ARG160-O6	0.7003
GLY13-O6	0.8001	THR68-O5	0.7634	MET165-C6	0.7249	CYS138-C10	0.7002
TYR210-O5	0.7996	LEU72-C6	0.7633	TRP58-C6	0.7241	PHE90-O6	0.7001
LYS65-ASP189	0.7979	VAL162-C6	0.7624	GLY184-C6	0.7235		
TYR61-C5	0.7976	LYS132-O5	0.7617	ASN5-C6	0.7234		
TYR61-O5	0.7965	ILE187-O6	0.7588	CYS138-C11	0.7233		
PRO149-C6	0.7929	TYR183-C6	0.7578	ARG160-C6	0.7221		
ALA62-O6	0.7924	THR68-C6	0.7546	SER161-O5	0.7197		
PHE213-O5	0.7919	MEt165-O55	0.7546	PHE90-O5	0.7196		
GLU131-O6	0.7905	VAL192-O5	0.7522	VAL192-C5	0.7191		

Supplementary Table 4. Descriptor correlation to tIC1 from feature-diverse tICA decomposition of *apo* transport.

System	APO
Feature	tIC1 correlation | *r* |
PHE43-PHE164	0.8442
PRO47-LEU169	0.8215
GLY42-PHE164	0.8017
PRO166-PRO47	0.7387
GLY42-GLU163	0.7355

Supplementary Table 5. Descriptor correlation to tIC2 from feature-diverse tICA decomposition of SUC transport.

System	SUC	System	SUC	System	SUC
Feature	tIC2 correlation | *r* |	Feature	tIC2 correlation | *r* |	Feature	tIC2 correlation | *r* |
ASN76-C2	0.8886	GLY16-C10	0.7623	LEU72-C10	0.7182
ASN76-O9	0.8760	PRO195-O9	0.7619	ASN17-O9	0.7180
ASN196-O9	0.8451	PRO195-C5	0.7615	SER20-C11	0.7160
ASN76-C9	0.8450	TRP180-O9	0.7607	SER142-O9	0.7157
ASN76-C11	0.8376	ASN17-C10	0.7597	TRP180-C2	0.7151
TRP58-C9	0.8302	SER54-C2	0.7595	ALA200-O6	0.7138
ASN196-C2	0.8292	PRO195-C2	0.7569	SER176-C5	0.7137
ASN76-C10	0.8189	ILE75-C10	0.7564	ASN196-O6	0.7128
TRP58-C10	0.8174	GLY16-C2	0.7561	SER142-C6	0.7120
ALA55-O9	0.8096	ASN196-O5	0.7558	SER176-C9	0.7112
ALA55-C2	0.8081	SER54-O9	0.7553	SER142-C2	0.7094
GLY79-C2	0.8003	SER20-C6	0.7537	LEU71-C9	0.7089
GLY79-O9	0.7986	ALA55-C9	0.7510	SER54-C11	0.7088
ASN76-C12	0.7936	GLY79-C11	0.7507	SER54-C9	0.7084
SER20-C2	0.7859	GLY199-O9	0.7506	SER20-O5	0.7082
SER20-O9	0.7852	ALA200-C6	0.7504	GLY16-C12	0.7080
TRP58-C2	0.7841	ASN17-C9	0.7488	GLY79-C6	0.7080
SER176-C2	0.7834	LEU72-C9	0.7483	GLY79-C12	0.7060
ASN196-C6	0.7826	PRO195-C6	0.7473	ASN17-C2	0.7052
ALA200-C2	0.7779	ALA200-C5	0.7457	SER176-C6	0.7043
ALA177-C2	0.7776	ILE75-C11	0.7430	ASN196-C11	0.7032
ILE75-C9	0.7773	ALA62-C10	0.7429	GLY79-C5	0.7010
ILE75-C2	0.7773	SER20-C5	0.7396	GLY79-C10	0.7001
ALA177-O9	0.7767	ASN17-C11	0.7387		
ALA200-O9	0.7755	ALA200-O5	0.7383		
GLY16-O9	0.7733	GLY199-C2	0.7364		
TRP58-C11	0.7717	GLY79-C9	0.7315		
GLY16-C11	0.7701	PRO195-O6	0.7296		
ASN196-C5	0.7696	ALA55-C11	0.7288		
GLY16-C9	0.7670	SER20-C10	0.7260		
SER176-O9	0.7664	SER20-C9	0.7246		
ILE75-O9	0.7639	TRP58-O9	0.7208		
ALA62-C9	0.7633	SER176-O6	0.7207		

Supplementary Table 6. Descriptor correlation to tIC2 from feature-diverse tICA decomposition of GLC transport.

System	GLC	System	GLC	System	GLC
**Feature**	**tIC2 correlation | *r* |**	**Feature**	**tIC2 correlation | *r* |**	**Feature**	**tIC2 correlation | *r* |**
ASN196-C3	0.8715	ILE75-C3	0.7969	SER20-O4	0.7482
ASN74-C4	0.8621	TYR61-O3	0.7960	VAL23-C3	0.7480
GLY79-C4	0.8550	GLY16-O3	0.7956	ASP189-O3	0.7439
TRP180-C4	0.8487	GLY184-C4	0.7952	ALA177-C4	0.7426
ASN76-O4	0.8487	VAL192-O3	0.7946	LEU72-C4	0.7424
ASN194-C4	0.8484	ALA200-C4	0.7939	ASN17-C4	0.7414
ASN194-O3	0.8484	ALA62-O4	0.7917	PHE24-O4	0.7393
ILE75-C4	0.8475	SER176-C3	0.7913	GLY199-C3	0.7393
TRP180-C4	0.8466	GLY16-C3	0.7909	VAL139-C4	0.7386
TRP58-C4	0.8380	VAL139-C3	0.7889	SER20-C3	0.7359
TRP58-C3	0.8375	TRP58-O3	0.7888	LEU72-O4	0.7353
ALA55-C4	0.8359	ALA200-O3	0.7884	GLY135-O3	0.7348
ASN76-C3	0.8354	PRO195-C4	0.7864	SER176-O3	0.7343
TRP58-O4	0.8335	PRO195-O3	0.7847	ILE75-O3	0.7336
TRP180-O3	0.8328	PHE24-C4	0.7845	LEU72-O3	0.7308
PRO195-C3	0.8319	VAL192-C3	0.7834	ASP189-C3	0.7279
GLY184-O3	0.8275	VAL139-O3	0.7803	ASN17-O4	0.7274
GLY16-C4	0.8268	TYR61-C4	0.7801	SER142-C4	0.7256
TYR183-O3	0.8244	VAL23-C4	0.7797	GLY79-O3	0.7253
GLY79-O4	0.8186	ALA62-C4	0.7784	GLY135-C3	0.7247
ILE75-O4	0.8168	ALA62-C3	0.7757	CYS138-C4	0.7232
GLY184-C3	0.8166	TYR183-O4	0.7730	CYS138-O4	0.7215
GLY79-C3	0.8152	SER142-C3	0.7708	SER54-C3	0.7204
ALA200-C3	0.8142	CYS138-C3	0.7702	PRO195-O4	0.7200
ALA55-C3	0.8119	ALA177-C3	0.7699	ALA55-O3	0.7179
SER20-C4	0.8101	CYS138-O3	0.7647	ALA200-O4	0.7164
GLY16-O4	0.8078	TRP180-O4	0.7644	ALA177-O3	0.7151
ALA55-O4	0.8073	ASN76-O3	0.7618	LEU72-O3	0.7141
TYR183-C3	0.8056	ALA62-O3	0.7601	SER54-C4	0.7141
TYR183-C4	0.8048	ASN196-O4	0.7544	VAL192-O4	0.7108
TYR61-C3	0.8033	PHE24-C3	0.7542	ASP189-C4	0.7043
TYR61-O4	0.8026	VAL192-C4	0.7531	VAL23-O4	0.7036
SER176-C4	0.7989	GLY184-O4	0.7524	VAL139-O4	0.7020

Supplementary Table 7. Realistic bilayer composition used during AtSWEET13 simulations.

	**Outer Leaflet**	**Inner Leaflet**
**Sterols**	68	29
β-sitosterol	46	20
Stigmasterol	22	9
	**Outer Leaflet**	**Inner Leaflet**
**Phospholipids**	36	67
PC	17	31
16:0/18:1 (POPC)	1	2
16:0/18:2 (PLPC)	9	17
18:2/18:2 (DLiPC)	4	7
18:2/18:3 (LLPC)	3	6
	**Outer Leaflet**	**Inner Leaflet**
PE	16	30
16:0/18:1 (POPE)	1	0
16:0/18:2 (PLPE)	10	19
18:2/18:2 (DLiPE)	3	6
18:2/18:3 (LLPE)	2	4
	**Outer Leaflet**	**Inner Leaflet**
PG	3	6
16:0/18:1 (POPG)	0	1
16:0/18:2 (PLPG)	3	5

Supplementary Table 8. Finalized MSM hyperparameters

System	# Clusters	tICA dimensions	tiCA lag time	MSM lag time
**APO**	950	3	8 ns	30 ns
**SUC**	900	8	8 ns	40 ns
**GLC**	750	12	8 ns	20 ns
