# Supplementary material for: The Arabidopsis AtSWEET13 transporter discriminates sugars by selective facial and positional substrate recognition
Source: Commun Biol. 2024 Jun 24;7:764. doi: 10.1038/s42003-024-06291-6 (PMC11196581; doi:10.1038/s42003-024-06291-6)
Supplement: Supplementary file 3 — Reporting Summary [file 42003_2024_6291_MOESM3_ESM.pdf]

nature portfolio

Corresponding author(s): Diwakar Shukla

Last updated by author(s): 04/09/2024

Reporting Summary

Nature Portfolio wishes to improve the reproducibility of the work that we publish. This form provides structure for consistency and transparency in reporting. For further information on Nature Portfolio policies, see our Editorial Policies and the Editorial Policy Checklist.

Statistics

For all statistical analyses, confirm that the following items are present in the figure legend, table legend, main text, or Methods section.

n/a	Confirmed	
☐	☒	The exact sample size (*n*) for each experimental group/condition, given as a discrete number and unit of measurement
☐	☒	A statement on whether measurements were taken from distinct samples or whether the same sample was measured repeatedly
☒	☐	The statistical test(s) used AND whether they are one- or two-sided<br>*Only common tests should be described solely by name; describe more complex techniques in the Methods section.*
☒	☐	A description of all covariates tested
☒	☐	A description of any assumptions or corrections, such as tests of normality and adjustment for multiple comparisons
☒	☐	A full description of the statistical parameters including central tendency (e.g. means) or other basic estimates (e.g. regression coefficient) AND variation (e.g. standard deviation) or associated estimates of uncertainty (e.g. confidence intervals)
☒	☐	For null hypothesis testing, the test statistic (e.g. *F*, *t*, *r*) with confidence intervals, effect sizes, degrees of freedom and *P* value noted<br>*Give P values as exact values whenever suitable.*
☒	☐	For Bayesian analysis, information on the choice of priors and Markov chain Monte Carlo settings
☒	☐	For hierarchical and complex designs, identification of the appropriate level for tests and full reporting of outcomes
☒	☐	Estimates of effect sizes (e.g. Cohen's *d*, Pearson's *r*), indicating how they were calculated

Our web collection on statistics for biologists contains articles on many of the points above.

Software and code

Policy information about availability of computer code

Data collection	Molecular dynamics (MD) simulations were performed using AMBER18.
Data analysis	System preparation was performed using the following software: PACKMOL 18.169, VMD 1.9.3, ParmEd 2.7.3, cpptraj 18.00 .<br>Simulation and structure file manipulations necessary for figure generation and analyses were performed using: cpptraj 18.00 .<br>Trajectory analyses were performed using: MDTraj 1.9.3, scikit-learn 0.24.2, and python codes utilizing the numpy library for python 3.6 .<br>Clustering analyses for adaptive sampling were performed using: MSMBuilder 3.8.0 .<br>Markov state model generation was performed using: PyEMMA 2.5.6 .<br>Representation of protein structures was performed using: UCSF Chimera 1.14 .<br>Analyses on individual protein snapshots was performed using: cpptraj 18.00, MDAnalysis 2.0.0, HOLE 2.2.005 .<br>Free energy landscape figures and other plots were generated using: Matplotlib 3.2.0 and jupyter notebooks .<br>All manuscript figures were assembled and exported to high resolution using: Adobe Illustrator 2021.

For manuscripts utilizing custom algorithms or software that are central to the research but not yet described in published literature, software must be made available to editors and reviewers. We strongly encourage code deposition in a community repository (e.g. GitHub). See the Nature Portfolio guidelines for submitting code & software for further information.

Data

Policy information about availability of data

All manuscripts must include a data availability statement. This statement should provide the following information, where applicable:

- Accession codes, unique identifiers, or web links for publicly available datasets
- A description of any restrictions on data availability
- For clinical datasets or third party data, please ensure that the statement adheres to our policy

Simulation and simulation-related results and python scripts; jupyter notebooks; MSM objects and PDBs of select AtSWEET13 conformational states are available at the Shukla Group github at the following web address: https://github.com/ShuklaGroup/AtSWEET13_sugars. Codes and files used in this manuscript have been made available. Codes used in this manuscript are provided in a Box link, which is included in the project description for the following github page. The link is embedded with public access privileges, meaning that clicking on the provided Box link will reveal access to the codes. Again, the Box link can be found from our github page: https://github.com/ShuklaGroup/AtSWEET13_sugars .

The Source Data for reproducing our figures, as well as all related statistical reporting, is included in our Dryad repository. The Dryad repository will be available at: 10.5061/dryad.8sf7m0cxn .

Research involving human participants, their data, or biological material

Policy information about studies with human participants or human data. See also policy information about sex, gender (identity/presentation), and sexual orientation and race, ethnicity and racism.

Reporting on sex and gender	NA
Reporting on race, ethnicity, or other socially relevant groupings	NA
Population characteristics	NA
Recruitment	NA
Ethics oversight	NA

Note that full information on the approval of the study protocol must also be provided in the manuscript.

Field-specific reporting

Please select the one below that is the best fit for your research. If you are not sure, read the appropriate sections before making your selection.

☒ Life sciences ☐ Behavioural & social sciences ☐ Ecological, evolutionary & environmental sciences

For a reference copy of the document with all sections, see nature.com/documents/nr-reporting-summary-flat.pdf

Life sciences study design

All studies must disclose on these points even when the disclosure is negative.

Sample size	NA
Data exclusions	NA
Replication	NA
Randomization	NA
Blinding	NA

Reporting for specific materials, systems and methods

We require information from authors about some types of materials, experimental systems and methods used in many studies. Here, indicate whether each material, system or method listed is relevant to your study. If you are not sure if a list item applies to your research, read the appropriate section before selecting a response.

Materials & experimental systems

n/a	Involved in the study
☒	☐ Antibodies
☒	☐ Eukaryotic cell lines
☒	☐ Palaeontology and archaeology
☒	☐ Animals and other organisms
☒	☐ Clinical data
☒	☐ Dual use research of concern
☒	☐ Plants

Methods

n/a	Involved in the study
☒	☐ ChIP-seq
☒	☐ Flow cytometry
☒	☐ MRI-based neuroimaging

Plants

Seed stocks	NA
Novel plant genotypes	NA
Authentication	NA
